# Click-Free Synthesis of a Multivalent Tricyclic Peptide as a Molecular Transporter

**DOI:** 10.3390/pharmaceutics12090842

**Published:** 2020-09-03

**Authors:** Sumit Kumar, Dindyal Mandal, Shaima Ahmed El-Mowafi, Saghar Mozaffari, Rakesh Kumar Tiwari, Keykavous Parang

**Affiliations:** 1 Center for Targeted Drug Delivery, Department of Biomedical and Pharmaceutical Sciences, Harry and Diane Rinker Health Science Campus, Chapman University School of Pharmacy, Irvine, CA 92618, USA; sumitkumarmalik@gmail.com (S.K.); dmandal@kiitbiotech.ac.in (D.M.); sa.el-mowafi@nrc.sci.eg (S.A.E.-M.); mozaf100@mail.chapman.edu (S.M.); 2 Department of Chemistry, Deenbandhu Chhotu Ram University of Science and Technology, Murthal, Haryana 131039, India; 3 School of Biotechnology, KIIT Deemed to be University, Bhubaneswar 751024, India; 4 Peptide Chemistry Department, National Research Centre, Dokki, Cairo 12622, Egypt

**Keywords:** antibacterial activity, cell-penetrating peptide, cyclic peptide, tricyclic, cellular uptake, cytotoxicity, drug delivery, MDA-MB-231, phosphopeptide, siRNA

## Abstract

The cellular delivery of cell-impermeable and water-insoluble molecules remains an ongoing challenge to overcome. Previously, we reported amphipathic cyclic peptides *c*[WR]_4_ and *c*[WR]_5_ consisting of alternate arginine and tryptophan residues as nuclear-targeting molecular transporters. These peptides contain an optimal balance of positive charge and hydrophobicity, which is required for interactions with the phospholipid bilayer to facilitate their application as a drug delivery system. To further optimize them, we synthesized and evaluated a multivalent tricyclic peptide as an efficient molecular transporter. The monomeric cyclic peptide building blocks were synthesized using Fmoc/tBu solid-phase chemistry and cyclization in the solution and conjugated with each other through an amide bond to afford the tricyclic peptide, which demonstrated modest antibacterial activity against methicillin-resistant *Staphylococcus aureus* (MRSA), *Klebsiella pneumoniae, Pseudomonas aeruginosa*, and *Escherichia coli* (*E. coli*) with a minimum inhibitory concentration (MIC) of 64–128 µg/mL. The tricyclic peptide was found to be nontoxic up to 30 µM in the breast cancer cell lines (MDA-MB-231). The presence of tricyclic peptide enhanced cellular uptakes of fluorescently-labeled phosphopeptide (F’-GpYEEI, 18-fold), anti-HIV drugs (lamivudine (F’-3TC), emtricitabine (F’-FTC), and stavudine (F’-d4T), 1.7–12-fold), and siRNA (3.3-fold) in the MDA-MB-231 cell lines.

## 1. Introduction

Multivalency is a pervasive phenomenon that has attracted attention for therapeutic development with the examples of multivalent aptamers, a multivalent effect in glycosidase inhibition, and many more [1,2,3,4]. Multivalent binding plays an essential role in signal transduction and self-organization in biological systems and is mainly based on the multiple simultaneous molecular recognition processes [5,6]. Nature’s one good example of multivalency is proteins forming multimeric architectures in the form of receptors or enzymes that contain several recognition sites.

Peptide-based multivalent interactions at interfaces of a biological system are of great importance. For example, the multivalency of carefully designed supramolecular peptide ligands was utilized in enhancing binding interaction with β-cyclodextrin (β-CD) to study host–guest relationships [7]. In another approach, multivalency was achieved using self-assembly of a coil/fiber-forming peptide to study the binding interaction with antibody and carbohydrate-binding motifs [8]. Henning et al. observed tenfold enhanced binding affinity of the lead peptide by generating multivalent property using dendritic polymer containing polyethylene glycol (PEG) against one of WW binding domains of FBP21 protein, which is important for splicing of pre-mRNA in the nucleus [9]. Using a very similar dendritic polymer with PEG scaffold, Lauster et al. developed multivalent peptide–nanoparticle conjugates for targeting spike protein on the influenza virus [10]. Multivalency is an important aspect, which needs more exploitation for other applications, such as molecular transporter and drug delivery, especially using peptide-based transporters.

**Figure 1:**
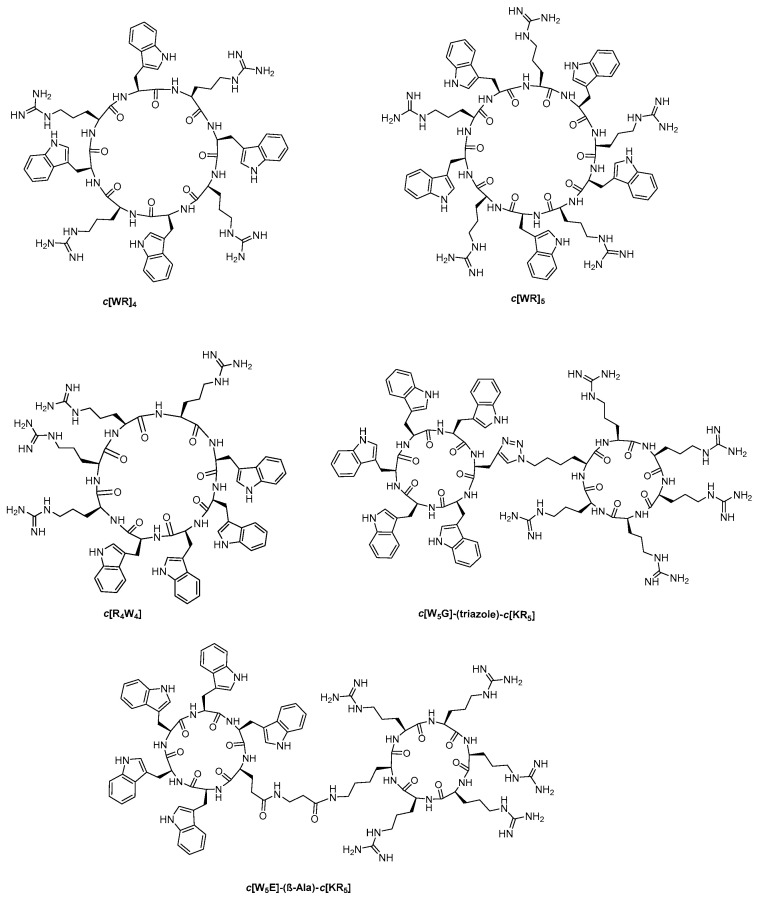
The chemical structures of previously reported mono- and bicyclic peptides, *c*[WR]_5_, *c*[WR]_4_ [16], *c*[R_4_W_4_] [23], and (*c*[W_5_G]-(triazole)-*c*[KR_5_], and *c*[W_5_E]-(β-Ala)-*c*[KR_5_]) [22].

Peptides are extensively used in drug delivery of a diverse class of therapeutics across phospholipid bilayer due to their cationic and amphipathic nature. Because of enhanced selectivity and metabolic stability of cyclic peptides, they are preferred to their counterpart linear peptides for drug delivery applications [11]. Besides several other classes of cyclic peptides [12,13,14,15], developed cyclic peptide *c*[WR]_4_ and *c*[WR]_5_ (Figure 1) [16] containing naturally occurring alternate L-tryptophan (W) and L-arginine (R) amino acid demonstrated a prominent application for the delivery of negatively-charged phosphopeptide, antiviral drugs, and anticancer drugs (doxorubicin, paclitaxel, and camptothecin) and were used as a capping agent to the selenium and gold nanoparticles for better drug delivery applications [17,18,19,20,21,22]. These series of cyclic peptides showed nuclear localization, low cytotoxicity, biodegradable property, and endocytosis-independent cellular uptake [13]. Thus, these properties made them excellent candidates as molecular transporters.

We also discovered that a block of four subsequent tryptophan and positive-charged arginine residues generated amphipathic antibacterial peptide *c*[R_4_W_4_], which demonstrated minimum inhibitory concentration (MIC) of 4 µg/mL against methicillin-resistant *Staphylococcus aureus* (MRSA) and 16 µg/mL against *E. coli* [23]. Furthermore, the structure-activity relationship showed the importance of tryptophan and arginine residues in this pattern for antibacterial activity [24].

Furthermore, to gain a deep understanding of *c*[WR]_5_ series of peptide for their molecular transporter property, we developed a series of bicyclic peptides (*c*[W_5_G]-(triazole)-*c*[KR_5_] and *c*[W_5_E]-(β-Ala)-*c*[KR_5_]) containing two cyclic peptides (*c*[W_5_G] and *c*[KR_5_]) joined through a flexible amide or nonflexible triazole linker (Figure 1) and evaluated the delivery of a negatively-charged phosphopeptide [22]. It was found that bicyclic peptide containing amide linkage *c*[W_5_E]-(β-Ala)-*c*[KR_5_] showed higher cellular uptake of a phosphopeptide as compared to monocyclic peptides (*c*[KR_5_] or *c*[WR]_4_). These data indicated that for better cellular uptake of these cyclic peptides, an optimal balance of positive charge and hydrophobicity is essential for interactions with the cell membrane and deep penetration into the lipid bilayer [15]. *c*[WR]_5_ consists of a balance of hydrophobicity and positive charges. We hypothesized that this peptide template could be extended into a tricyclic peptide platform as a multivalent structure for application in drug delivery.

Most of the biological interactions are controlled via multivalent interactions with respect to density, spacing, complex interaction, and various dynamic effects [25,26,27,28]. Thus, it is always challenging to effectively be amended. Multivalent constructs were prepared using preorganized cyclopeptides or dendrimers using various methodologies, mainly “click reactions” [2]. Copper-catalyzed “click reaction” has various advantages, but the introduction of copper species to in vivo systems raises the concern of potential toxicity [29,30,31]. Various strategies, including the development of chelating azides as reagents, are being adopted to reduce the copper-induced toxicity [32]. We have previously shown that higher cellular uptake of a phosphopeptide in the presence of bicyclic peptide containing amide linkage *c*[W_5_E]-(β-Ala)-*c*[KR_5_] than bicyclic peptide (*c*[W_5_G]-(triazole)-*c*[KR_5_] [22], presumably due to the more flexibility of the linker between two cyclic peptides containing the amide linker for generating a proper conformation for effective interaction with the cell membrane.

Thus, to unravel a new multivalent tricyclic peptide as a molecular transporter, herein, we synthesized a tricyclic peptide based on naturally occurring amino acids using a simpler biocompatible and biodegradable amidic linkage instead of a triazole formed through click chemistry. The tricyclic peptide was a trimer of monocyclic peptides containing alternating hydrophobic residues (W) and charged residues (R) in addition to lysine (K) residues that assist in the generation of an amide linkage through their side chain amino group. Herein, we report antibacterial activity and molecular transporter properties of the tricyclic peptide.

## 2. Materials and Methods

### 2.1. Materials

Fmoc amino acids, coupling reagents, and amino acid-loaded chlorotrityl resin were obtained from AAPPTec (Louisville, KY, USA). The solvents and chemical reagents were purchased from MilliporeSigma (Milwaukee, WI, USA) and used without further purification. Crude peptides were purified using reverse phase high performance liquid chromatography (RP-HPLC) using Hitachi L-2455 system (Canby, OR, USA) with a C18 Phenomenex column (Prodigy, 10 µm, 2.1 cm × 25 cm), at a flow rate of 10 mL/min with the detection at 214 nm using a gradient of 0–100% acetonitrile (0.1% trifluoracetic acid (TFA)) and water (0.1% TFA) over 60 min. Peptides were characterized for confirmation of their exact mass using Bruker GT 0264 (Fremont, CA, USA) matrix-assisted laser desorption/ionization (MALDI) mass spectrometer with α-cyano-4-hydroxycinnamic acid as a matrix under positive mode. Human breast cancer (MDA-MB-231, HTB-26) cell line was obtained from American Type Culture Collection (ATCC, Manassas, VA, USA). Cell culture media were obtained from Fisher Scientific. Bacterial strains: Methicillin-resistant *Staphylococcus aureus* (MRSA) (Los Angeles County (LAC) clone) and *E. coli* (ATCC 25922) were acquired from the Loss Angeles Public Health Department (Los Angeles, CA, USA). *Pseudomonas aeruginosa* (ATCC 27883) and *Klebsiella pneumoniae* (ATCC BAA 1705) were obtained from ATCC. The media for bacterial experiments were purchased from Hardy diagnostic (Santa Maria, CA, USA).

### 2.2. Chemistry

#### 2.2.1. General Method for the Synthesis of Monocyclic Peptides

In general, all cyclic peptides were synthesized by the Fmoc solid-phase synthesis strategy followed by cyclization in the solution. H-Arg(Pbf)-2-chlorotrityl resin (**1**) (1 g, 0.58 mmol, 0.58 meq/g, 100–200 mesh) was swelled in *N*,*N*-dimethylformamide (DMF) (50 mL) under nitrogen in a 500 mL peptide synthesis vessel. After 2 h, DMF was drained out, and a solution of Fmoc-Trp(Boc)-OH (1.74 mmol, 916.3 mg), 1-[bis(dimethylamino)methylene]-1*H*-benzotriazolium hexafluorophosphate 3-oxide (HBTU) (1.74 mmol, 660 mg), and *N,N*-diisopropylethylamine (DIPEA) (3.48 mmol, 606 µL) in DMF (20 mL) was added to the resin. The resin was agitated under nitrogen for 30 min for coupling the first amino acid. After 30 min, the reaction mixture was drained and washed with DMF (10 mL × 3 times). A solution (20 mL) of 20% piperidine in DMF (*v/v*) was added to remove the *N*-terminal Fmoc group with the agitation of peptidyl resin for 20 min two times followed by washing with DMF (10 mL × 3 times). In a similar way, the subsequent Fmoc-protected amino acids were coupled based on the required sequence of the linear peptides. Once the linear protected peptide was assembled on the peptidyl resin, the side-chain-protected peptide was cleaved from the resin for *N* to *C* terminal cyclization. The peptidyl resin was stirred with freshly prepared cleavage cocktail containing trifluoroethanol (TFE)/acetic acid/dichloromethane (DCM) (2:1:7, *v/v/v*, 50 mL) for 2 h. The resin was filtered off, and the supernatant was evaporated to dryness to get side chain-protected linear peptides (**2**, **8**, **13**). The side-chain-protected peptide (300 mg) was taken in 2 L round bottom flask under nitrogen. DMF and DCM (2:1, *v/v*, 1.2 L) were added to the mixture. A solution of 1-hydroxyazabenzotriazole (HOAt) (236.8 mg) in DMF and *N,N’*-diisopropylcarbodiimide (DIC) (269.4 µL) were added. The reaction was stirred for 24 h under nitrogen. The solvents were evaporated under vacuum, and side chains were deprotected by agitation with freshly prepared cleavage cocktail containing TFA/thioanisole/anisole/1,2-ethanedithiol (EDT) (90:5:2:3, *v/v/v/v*, 10 mL) for 2 h. The crude peptides were precipitated by the addition of cold diethyl ether (Et_2_O) and centrifugation. Crude peptides were purified with RP-HPLC. The peptides were separated by eluting the crude peptides at 10.0 mL/min using a gradient of 0–100% acetonitrile (0.1% TFA) and water (0.1% TFA) over 60 min and then characterized by MALDI TOF mass spectroscopy. The desired fractions were pooled and lyophilized to yield cyclic peptides. MALDI spectra of intermediate and synthesized compounds and selected analytical HPLC have been provided in the Supplementary Materials.

#### 2.2.2. Synthesis of Monocyclic Cyclic Peptides Containing Free Amino or Free Carboxylic Acid or Both

[D-W(Boc)-R(Pbf)-W(Boc)-R(Pbf)-K(Dde)-W(Boc)-R(Pbf)-W(Boc)-R(Pbf)] (**4**) containing free carboxylic acid.

The side-chain-protected linear peptide (NH_2_-D(OAll)-W(Boc)-R(Pbf)-W(Boc)-R(Pbf)-K(Dde)-W(Boc)-R(Pbf)-W(Boc)-R(Pbf)-tritryl resin) was assembled on H-Arg(Pbf)-2-chlorotrityl resin (**1**, 1 g, 0.58 mmol) using building blocks of Fmoc-protected amino acids, such as Fmoc-Trp(Boc)-OH (916.3 mg), Fmoc-Arg(Pbf)-OH (1128 mg), Fmoc-Lys(Dde)-OH (926.7 mg), and Fmoc-Asp-(OAll)-OH (687.9 mg), followed by cleavage from the solid support to afford 460 mg (24%) of the linear side-chain-protected peptide (NH_2_-D(OAll)-W(Boc)-R(Pbf)-W(Boc)-R(Pbf)-K(Dde)-W(Boc)-R(Pbf)-W(Boc)-R(Pbf)-COOH) (**2**). MALDI-TOF (*m/z*) for **2**, [C_91_H_124_N_27_O_15_]: calcd, 1834.9765; found, 1835.1590 [M + H]^+^.

Compound **2** underwent cyclization to obtain side-chain-protected peptide [D(OAll)-W(Boc)-R(Pbf)-W(Boc)-R(Pbf)-K(Dde)-W(Boc)-R(Pbf)-W(Boc)-R(Pbf)] (**3**) using general peptide synthesis method explained in Section 2.2.1. Cyclic peptide **3**, 418 mg (91%) was dried for 24 h before using for the subsequent reaction. MALDI-TOF (*m*/*z*) for **3**, [C_91_H_121_N_27_O_14_]: calcd, 1815.9586; found, 1817.9611 [M + 2]^+^.

To obtain compound **4**, the protected cyclic peptide **3** (200 mg) was taken in a dry round bottle flask fitted with a rubber septum and under the inert condition with N_2_. A solution containing Pd(PPh_3_)_4_ (2.01 g, 0.00174 mmol, 3.0 equiv) and CHCl_3_:AcOH:*N*-methyl morpholine (37:2:1, *v/v/v*, 25 mL) was added, and the mixture was stirred for 2 h followed by evaporation of solution under rotatory evaporator. A solution of 0.5% DIPEA and sodium diethyldithiocarbamate (0.5% *w/w*) in DMF was added to the evaporated residue to remove the catalyst. The solvent was evaporated to remove all the solvents. The crude mixture was washed with water (8–10 mL) to get rid of all side products following with centrifugation (1200 rpm, 3 × 5 min). After centrifuging the crude peptide, it was dried overnight to obtain the protected cyclic peptide **4** (136 mg, 69%). MALDI-TOF (*m*/*z*) for **4**, [C_88_H_117_N_27_O_14_]: calcd, 1775.9273; found, 1777.8700 [M + 2]^+^.

[D(OAll)-W(Boc)-R(Pbf)-W(Boc)-R(Pbf)-K-W(Boc)-R(Pbf)-W(Boc)-R(Pbf)] (**5**) containing free amino group.

To obtain the Dde-deprotected cyclic peptide **5**, a solution of 2% hydrazine monohydrate solution in DMF (50 mL) with 7.8 mL of allyl alcohol was added to the peptide **3** (200 mg) and stirred at room temperature for 2 h. The reactant mixture was evaporated under vacuum to dryness and washed with cold water. The obtained solid was dried to yield side-chain-protected cyclic peptide **5** (124 mg, 65%). MALDI-TOF (*m*/*z*) for **5**, [C_81_H_111_N_27_O_12_]: calcd, 1653.8895; found, 1654.5044 [M + H]^+^.

Deprotected cyclic peptide [DWRWRKWRWR] (**7**) containing free amino and free carboxylic acid.

Peptide **4** was deprotected using 2% hydrazine monohydrate in DMF to afford protected cyclic peptide [DW(Boc)R(Pbf)W(Boc)R(Pbf)KW(Boc)R(Pbf)W(Boc)R(Pbf)] (**6**). Alternatively, peptide **5** was deprotected using Pd(PPh_3_)_4_ and CHCl_3_:AcOH:*N*-methylmorpholine as mentioned above to obtain **6** (94 mg, ~72% + 90 mg, 74%). Finally, peptide **6** was completely deprotected to remove Pbf and Boc groups by stirring with freshly prepared cleavage cocktail containing TFA/thioanisole/anisole/1,2-ethanedithiol (EDT) (90:5:2:3, *v/v/v/v*, 10 mL) for 2 h to yield fully deprotected cyclic peptide [DWRWRKWRWR] **7**. MALDI-TOF (*m*/*z*) for **7**, [C_78_H_105_N_27_O_12_]: calcd, 1611.8436; found, 1613.2794 [M + H]^+^.

#### 2.2.3. Synthesis of Diamine Cyclic Peptide [KWRWRKWRWR] (**10**) and Diacid Cyclic Peptide [K(COCH_2_CH_2_COOH)WRWRK(COCH_2_CH_2_COOH)WRWR] (**12**)

Using the general protocol mentioned in Section 2.2.1, the monocyclic peptide was assembled on H-Arg(Pbf)-2-chlorotrityl resin (**1**, 1 g, 0.58 mmol) using building blocks of Fmoc-protected amino acids, such as Fmoc-Trp(Boc)-OH (916.3 mg), Fmoc-Arg(Pbf)-OH (1128 mg), and Fmoc-Lys(Dde)-OH (926.7 mg), based on the sequence of amino acids in the diamine cyclic peptide **10** followed by cleavage from solid support to afford side-chain-protected linear peptide NH_2_-K(Dde)-W(Boc)-R(Pbf)-W(Boc)-R(Pbf)-K(Dde)-W(Boc)-R(Pbf)-W(Boc)-R(Pbf)-COOH (**8**, 432 mg, 22%). MALDI-TOF (*m*/*z*) for **8**, [C_100_H_138_N_28_O_15_]: calcd, 1971.0896; found, 1972.3850 [M + H]^+^.

Compound **8** was cyclized and deprotected for Dde group using HOAt/DIC and 2%hydrazine in DMF, respectively, to provide side-chain-protected cyclic peptide [K-W(Boc)-R(Pbf)-W(Boc)-R(Pbf)-K-W(Boc)-R(Pbf)-W(Boc)-R(Pbf)] (**9**, 321 mg, 89%). Cyclic peptide **9** was divided into two parts (120 mg and 200 mg). 

To the first part, a freshly prepared cleavage cocktail containing TFA/thioanisole/anisole/1,2-ethanedithiol (EDT) (90:5:2:3, *v/v/v/v*, 10 mL) was added. The mixture was stirred at room temperature for 2 h to fully deprotect side chain protecting groups and afford **10**. MALDI-TOF (*m*/*z*) for **10**, [C_80_H_112_N_28_O_10_]: calcd, 1624.9116; found, 1626.4005 [M + H]^+^.

To the second part of peptide **9** (200 mg) taken in DCM (25 mL), triethylamine (242.5 µL) was added at 0 °C followed by the addition of succinic anhydride (174.1 mg). The mixture was stirred at room temperature for 2 h. DCM was evaporated under vacuum, and the solid residue was washed with water and dried to afford crude product [K(COCH_2_CH_2_COOH)-W(Boc)-R(Pbf)-W(Boc)-R(Pbf)-K(COCH_2_CH_2_COOH)W(Boc)R(Pbf)W(Boc)R(Pbf)] (**11**, 210 mg, 98%). After drying the crude product, the side chain-protecting groups were deprotected by stirring peptide **11** with a freshly prepared cleavage cocktail containing TFA/thioanisole/anisole/1,2-ethanedithiol (EDT) (90:5:2:3, *v/v/v/v*, 10 mL) at room temperature for 2 h. The peptides were precipitated and centrifuged to obtain a fully deprotected diacid of cyclic peptide [K(COCH_2_CH_2_COOH)-WRWR-K(COCH_2_CH_2_COOH)WRWR] (**12**). MALDI-TOF (*m*/*z*) for [K(COCH_2_CH_2_COOH)-WRWR-K(COCH_2_CH_2_COOH)WRWR] (**12**), [C_88_H_120_N_28_O_16_]: calcd, 1824.9437; found, 1826.3790 [M + H]^+^.

#### 2.2.4. Synthesis of a Monoamine Cyclic Peptide [RWRWKRWRW] (**16**) and a Mono Carboxylic Acid Cyclic Peptide [K(COCH_2_CH_2_COOH)-WRWRWRWR] (**18**)

Using the general protocol mentioned in Section 2.2.1, the monocyclic peptide was assembled on H-Arg(Pbf)-2-chlorotrityl resin (**1**, 1 g, 0.58 mmol) using building blocks of amino acids, Fmoc-Trp(Boc)-OH (916.3 mg), Fmoc-Arg(Pbf)-OH (1128 mg), and Fmoc-Lys(Dde)-OH (926.7 mg) based on the sequence of peptide **16**. The linear assembled peptide (**13**, 428 mg, 24%) was cleaved from the solid support and cyclized as mentioned in the general protocol to obtain crude cyclized peptide [K(Dde)-W(Boc)-R(Pbf)-W(Boc)-R(Pbf)-W(Boc)-R(Pbf)-W(Boc)-R(Pbf)] (**14**, 377 mg, 88%).

To peptide **14** (300 mg), a solution of 2% hydrazine monohydrate in DMF (50 mL) was added to deprotect Dde group. The solution was stirred for 2 h, and the reaction mixture was evaporated under vacuum to dryness and washed with cold water. The solid obtained was dried to obtain side-chain-protected cyclic peptide [K-W(Boc)-R(Pbf)-W(Boc)-R(Pbf)-W(Boc)-R(Pbf)-W(Boc)-R(Pbf)] (**15**, 354 mg, 93%). Peptide **15** was divided into two parts (154 mg and 200 mg).

To the first part of peptide **15** (154 mg), a freshly prepared cleavage cocktail containing TFA/thioanisole/anisole/1,2-ethanedithiol (EDT) (90:5:2:3, *v/v/v/v*, 10 mL) was added and stirred at room temperature for 2 h to deprotect side chain-protecting group. The second part of peptide **15** (200 mg) was dissolved in DCM (25 mL) and kept at 0 °C. Triethylamine (121.3 µL) was added to the solution of succinic anhydride (87.1 mg) in DCM was added slowly to the reaction mixture at 0 °C. The reaction mixture was stirred at room temperature for 2 h. DCM was evaporated under vacuum and the residue was washed with water and dried to obtain crude product [K(COCH_2_CH_2_COOH)-W(Boc)-R(Pbf)-W(Boc)-R(Pbf)-W(Boc)-R(Pbf)-W(Boc)-R(Pbf)] (**17**, 196 mg, 98%). The side chain protecting group of peptide **17** was deprotected by reacting with freshly prepared cleavage cocktail containing TFA/thioanisole/anisole/1,2-ethanedithiol (EDT) (90:5:2:3, *v/v/v/v*, 10 mL) at room temperature for 2 h.

The cleaved peptides were precipitated and centrifuged to obtain fully deprotected cyclic peptides containing monoamine [KWRWRWRWR] (**16**) and monocarboxylic acid [K(COCH_2_CH_2_COOH)-WRWRWRWR] **(18)**. MALDI-TOF (*m*/*z*) for [KWRWRWRWR] (**16**), [C_74_H_101_N_26_O_9_]: calcd, 1496.8167; found, 1497.7751 [M + 1]^+^. MALDI-TOF (*m*/*z*) for [K(COCH_2_CH_2_COOH)-WRWRWRWR] (**18**), [C_78_H_106_N_26_O_12_]: calcd, 1596.8237; found, 1598.7387 [M + 2]^+^.

#### 2.2.5. Synthesis of Tricyclic Peptide **20**

To a round bottom flask, the side-chain-protected cyclic peptides, diamine peptide (**9**) (10.0 mg, 3.29 × 10^−6^ mol) and monocarboxylic acid peptide (**17**) (39.6 mg, 13.18 × 10^−6^ mol) were added in 10 mL anhydrous DMF under stirring and inert condition using septum and purge of N_2_. The reaction mixture was kept at 0 °C followed by slow addition of *N*-ethyl-*N′*-(3-dimethylaminopropyl)carbodiimide hydrochloride (EDC.HCl) (3.1 mg, 16.47 mmol) and 4-dimethyl aminopyridine (DMAP) (0.2 mg, 1.65 mmol) with stirring for 1 h. The reaction mixture was allowed to reach room temperature and stirred for 24 h. The completion of the reaction was monitored by mass analysis of aliquot after complete deprotection of the reaction mixture using MALDI. Once the reaction was completed, the reaction mixture was evaporated to the dryness to yield peptide **19**, 24 mg, 80%, and the residue was treated with the freshly prepared cleavage cocktail containing TFA/thioanisole/anisole/1,2-ethanedithiol (EDT) (90:5:2:3, *v/v/v/v*, 10 mL) at room temperature for 2 h. The peptide was precipitated and centrifuged to obtain fully deprotected tricyclic peptide **20**, crude 10 mg, 78% that was purified by HPLC as described above to get pure compound 6 mg. MALDI-TOF (*m*/*z*) for **20**, [C_236_H_316_N_80_O_32_]: calcd, 4782.5559; found, 4782.1199 [M]^+^.

### 2.3. Bioassay

#### 2.3.1. Antibacterial Assay

The selected strains of bacteria, MRSA (Los Angeles County (LAC) clone), *E. coli* (ATCC 25922), *Pseudomonas aeruginosa* (ATCC 27883), and *Klebsiella pneumoniae* (ATCC BAA 1705) were cultured according to the guidelines of the Clinical Laboratory Standards Institute (CLSI) guidelines. MIC values were determined using microbroth dilution assay. Tricyclic peptide **20** was used to evaluate antibacterial activity in comparison to positive controls, such as Meropenem for Gram-negative bacteria and *c*[R_4_W_4_] for Gram-positive bacteria. Bacterial strains were inoculated into LB (5 mL) and shaken in an orbital shaker at 175 rpm overnight at 37 °C. The cultured suspension was immediately diluted to get turbidity of 0.5 McFarland (1.5 × 10^8^ bacterial cell CFU/mL) using normal saline. The test compounds (tricyclic peptide **20**, Meropenem, and *c*[R_4_W_4_]) were dissolved in 5% dimethyl sulfoxide (DMSO) to generate 256 µg/mL solutions. 200 µL of tricyclic peptide **20**, and controls were added to the first column of the 96 well plates, and a twofold serial dilution was performed with the addition of 100 µL of bacterial culture media to the other wells. The corresponding control DMSO 5% alone without the compound in similar concentration exhibited no antibacterial activity when diluted in the final concentration. Bacterial solution (100 µL) was added to all the wells and incubated statically at 37 °C overnight. The minimum concentration at which bacterial growth was not visible to eyes were reported as MICs and experiments were performed in triplicate.

#### 2.3.2. Cellular Cytotoxicity Assay

Human breast cancer (MDA-MB-231) cell line was grown on 75 cm^2^ cell culture flasks with Dulbecco’s modified eagle’s medium (DMEM) medium, supplemented with 10% fetal bovine serum (FBS), and 1% penicillin-streptomycin solution (10,000 units of penicillin and 10 mg of streptomycin in 0.9% NaCl) in a humidified atmosphere of 5% CO_2_, 95% air at 37 °C. CellTiter 96^®^ Aqueous (Promega, Madison, WI, USA) one solution cell proliferation assay (MTS) was utilized to evaluate MDA-MB-231 cell viability. Cells were seeded into 96-well plate (10^4^ cells/100 μL) in complete media and incubated overnight at 37 °C with 5% CO_2_. Different concentrations (0−300 μM) of the peptide solution (10 μL) were added to wells to make the final concentrations of the peptide (0−30 μM). The cells were incubated again (37 °C, 5% CO_2_) for 24 h, then MTS reagent (20 μL) was added to each well followed by incubation for 2 h under the same condition. The absorbance of the resulting solution was evaluated at 490 nm using a microplate reader to detect the live cells. Cells without peptide were considered as a control. DMSO (30% *v/v*) was used as a positive control. The compound was used in aqueous solution. All the tests were performed in triplicate. 

#### 2.3.3. Cellular Uptake of Fluorescence-Labeled Phosphopeptide (F’-GpYEEI) and Anti-HIV Drugs (Lamivudine (F’-3TC), Emtricitabine (F’-FTC), and Stavudine (F’-d4T)

Human breast cancer cells (MDA-MB-231) were grown in 6-well plates (2 × 10^5^ cells/well) in DMEM overnight. Then different fluorescence-labeled molecules (F’-3TC, F’-FTC, and F’-d4T) at 5 μM or F’-GpYEEI (5 μM) in the presence or absence of the tricyclic peptide **20** (25 μM) in opti-MEM were added, where F’ represents carboxyfluorescein and pY represents the phosphotyrosine amino acid). F’-GpYEEI, F’-3TC, F’-FTC, or F’-dT4 and cyclic peptide **20** were premixed in opti-MEM at room temperature for 30 min. The cells were incubated with the premixed solution for 3 h at 37 °C. 5-(6) carboxyfluorescein (FAM or F’) was used as a negative control. After 3 h incubation, the media containing peptide was removed. The cells were washed twice with PBS, followed by digestion with 0.25% trypsin/0.53 mM EDTA for 5 min to remove any false surface binding. Then PBS was added to wash the cells. The cells were centrifuged (600 rpm, 5 min) to collect the pellet. Finally, the obtained pellet was resuspended in buffer designated for flow cytometry and analyzed by flow cytometry (FACSCalibur: Becton, Dickinson & Co. (Franklin Lakes, NJ, USA)) using FITC channel and CellQuest software. The data depicted in the results are determined by using the mean fluorescence signal for 10,000 cells collected. Flow cytometry analysis was performed in triplicate.

#### 2.3.4. siRNA Delivery

Peptide/siRNA complexes were made by simple mixing of fluorescence-labeled siRNA (FAM-siRNA, Qiagen, Cat no 1027292) and peptides in different N/P ratios. First, FAM-siRNA was added to 150 mM NaCl solution. Tricyclic peptide **20** was dissolved in sterilized water to make a stock concentration of 1 mM. The peptide solution was mixed gently with siRNA/saline mixture at different N/P ratios. Then the resulting mixtures were allowed to stand for 30 min at room temperature for complete complex formation. N/P ratio was determined by the following equation:

N/P = (Number of moles for peptide × Number of Nitrogens)/(Number of moles for siRNA × 48) where 48 represents the average number of phosphates in each siRNA molecule.

Cells were seeded in 24-well plates at ~200,000 cells/mL. After overnight seeding, peptide/siRNA complexes were added. Peptide/siRNA complexes were made with peptide: siRNA N/P ratios of 10, 20, and 40, respectively. After 24 h of incubation at 37 °C, the cells were washed with hanks balanced salt solutions (HBBS), trypsinized, and fixed with 3.7% formaldehyde solution. Quantification of siRNA uptake was performed by Flow cytometer (BD-FACSVerse, BD Biosciences; San Jose, CA, USA) using the FITC channel to evaluate intracellular fluorescence signal. The mean fluorescence of cells in the selected cell population was determined. The results were calibrated by gating with the control cells (i.e., “No Treatment”) group in such a way that the auto-fluorescent cells became ~1% of the total cell population.

#### 2.3.5. Statistical Analyses

To evaluate the statistical significance of the results, ANOVA test was carried out using a single factor in MS Excel 2016. In case of cytotoxicity assay, cells without treatment were considered as the control and compared with the treated groups. For cellular uptake studies, only drug treated cells were considered as the control and compared with drug/carrier mixture. *p*-value less than 0.05 was considered as statistically significant and included in the Figures.

## 3. Results & Discussion

### 3.1. Chemistry

**Scheme 1:**
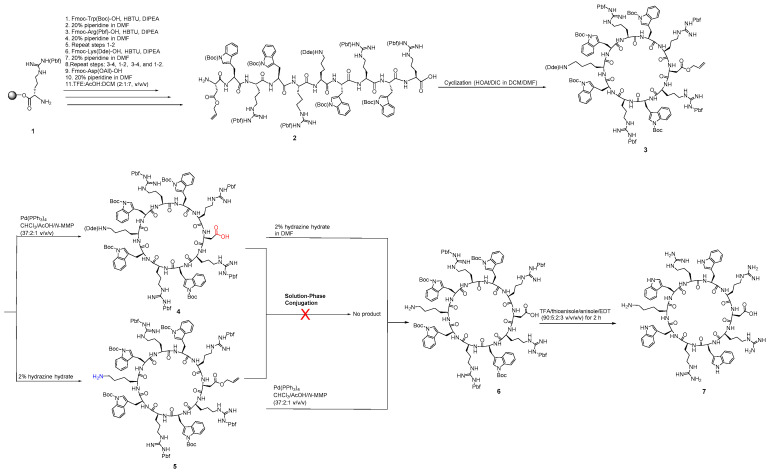
Synthesis of monocyclic peptides containing free amino or free carboxylic acid or both.

For the synthesis of click-free tricyclic peptide, we designed and followed various strategies. In the first approach, we started with the synthesis of two cyclic peptides that contained one free carboxylic acid and one free amino group to perform intermolecular conjugation between them or to oligomerize a cyclic peptide containing a free amino group and a free carboxylic acid [33]. Scheme 1 depicts the synthesis of monocyclic peptides and efforts to perform conjugation to develop bicyclic or tricyclic peptides. The exterior square bracket [] is used to represent cyclic peptides obtained through *N* to *C* terminal cyclization; however, parentheses () represent linear peptides.

The side-chain-protected linear peptide (**2**) was assembled on 2-chlorotrityl resin starting from H-Arg(Pbf)-2-chlorotrityl resin (**1**) using Fmoc/tBu solid-phase peptide synthesis strategy followed by cleavage of the protected peptide from the resin using cleavage cocktail (TFE:DCM:AcOH) at the room temperature to afford **2**. Linear protected peptide **2** was cyclized under dilute condition using HOAt and DIC to yield protected peptide **3** according to the previously reported procedure [16]. Cyclic peptide **3** contains an orthogonal protecting group of lysine known as Dde (4,4-dimethyl-2,6-dioxocyclohex-1-ylidene)ethyl) at the epsilon amino group of the lysine side chain. The Dde was selectively deprotected in the presence of 2% hydrazine monohydrate solution in DMF to generate a cyclic peptide containing a free NH_2_ group in (**5**).

While to get the cyclic peptide with the free carboxylic acid, the orthogonal protecting group of allyl ester of aspartic acid (OAll) in cyclic peptide **3** was selectively deprotected under a mild condition and Pd(0) catalyst to afford (**4**). Cyclic peptides **4** and **5** were reacted in the presence of different coupling reagents, but the conjugation reaction was not successful, possibly due to the steric hindrance of bulky groups present in both peptides.

Alternatively, the cyclic peptides **4** and **5** were further deprotected for removal of orthogonal protecting groups using 2% hydrazine monohydrate solution in DMF and tetrakis(triphenylphosphine)palladium(0) under mild condition, respectively, to afford cyclic peptide **6**, which was completely deprotected to remove Pbf and Boc groups to afford bifunctional cyclic peptide [DWRWRKWRWR] (**7**) containing both free amino and carboxylic acid groups as new bifunctional cyclic peptide. The conjugation of the cyclic peptide with itself failed to generate any dicyclic peptide.

**Scheme 2:**
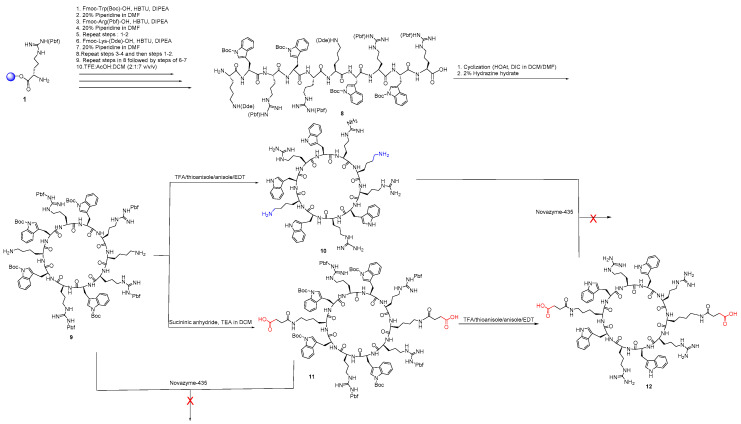
Synthesis of cyclic peptides containing two free amino or two free carboxylic acids.

In the second approach, we attempted to explore the enzymatic condensation polymerization between a diamine functional group-containing cyclic peptide **10** and a diacid functional group-containing cyclic peptide **12** using Novazyme-435, a lipase usually used for polyamide or polyester synthesis [34,35]. Thus, we included two lysine amino acid residues in the sequence of the cyclic peptide to get diamine residue, which could also be further modified from intermediate protected peptide **9** to diacid **12** after reacting to succinic anhydride. Scheme 2 depicts the synthesis of cyclic peptide building blocks (**10** and **12**).

The synthesis was started with the synthesis of side-chain-protected linear peptide **8** followed by cyclization and deprotection of Dde groups to yield side-chain-protected cyclic peptide **9**. Peptide **9** was divided into two parts, and one part was completely deprotected in the side chains to afford cyclic peptide containing diamine functional group [KWRWRKWRWR] **10**. The second part of cyclic peptide **9** was reacted with succinic anhydride in anhydrous condition to obtain a diacid functionalized side-chain-protected peptide **11**. The complete deprotection afforded diacid containing two free carboxylic acids in the cyclic peptide (**12**).

The enzymatic synthesis of multivalent cyclic peptide or polymerization of peptide **9** with peptide **11** and peptide **10** with peptide **12** were attempted using Novazyme-435 as a catalyst at 50 °C, 60 °C, and 70 °C without success, presumably due to lack of access of the cyclic peptides to the catalytic triad of the enzyme because of the bulky size.

**Scheme 3:**
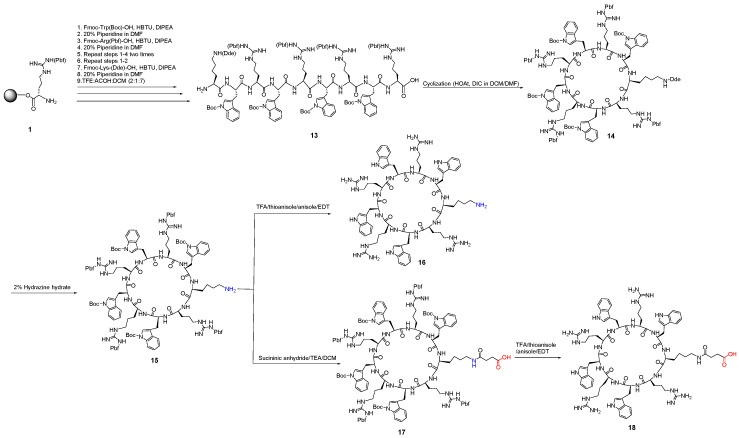
Synthesis of cyclic peptides containing one free amino group (**16**) and one free carboxylic acid (**17** and **18**).

In the third approach, we designed and synthesized cyclic peptides with a free monoamine functional group (**16**) and free monocarboxylic acid functional group (**18**) as shown in Scheme 3. The protected linear peptide (**13**) was synthesized, followed by cyclization to afford cyclic peptide **14**. The Dde group was removed using hydrazine to afford protected peptide **15**, which was divided into two parts. The first part underwent complete deprotection to yield mono amine-containing cyclic peptide [KWRWRWRWR] (**16**). The second part was functionalized by reacting with succinic anhydride to afford mono acid-containing cyclic peptide **17**. The complete deprotection of peptide **17** afforded mono carboxylic acid-containing cyclic peptide **18**.

**Scheme 4:**
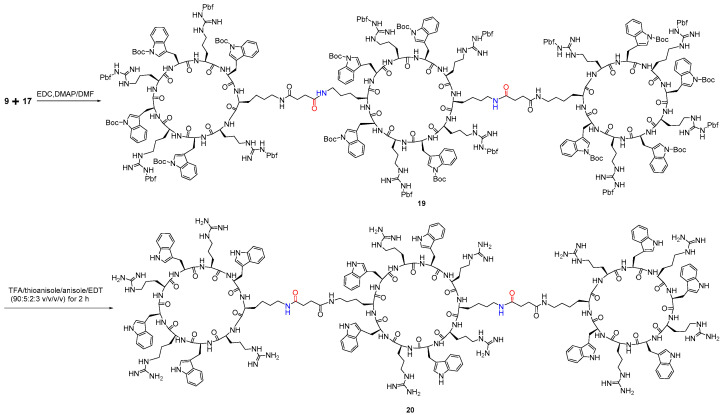
Synthesis of tricyclic peptide **20** from monomeric cyclic peptides **9** and **17**.

Finally, the conjugation of monocarboxylic containing cyclic peptide **17** was performed with diamine cyclic peptide **9** using EDC/DMAP as coupling reagents under an anhydrous condition for 24 h to obtain side-chain-protected tricyclic peptide **19** (Scheme 4). The complete deprotection of side chains in the tricyclic peptide **19** yielded multivalent tricyclic peptide **20**. The purity of the peptide was confirmed by analytical HPLC.

The analysis of our synthetic effort reflects that conjugation or coupling of a bifunctional cyclic peptide containing protected side chains or unprotected cyclic peptide (first approach) was not successful due to the steric hindrance associated with the side chains of tryptophan residues in the cyclic peptide. Moreover, steric hindrance may be due to the use of carboxylic acid for conjugation from the aspartic acid side chain, which was in the proximity of the backbone of the cyclic peptide. Therefore, we tried to use a lipase family enzyme named Novazyme-435 to facilitate conjugation and or polymerization of diamine or diacids containing protected or unprotected side chains in the cyclic peptides. However, the MALDI analysis did not show any conjugation. Novazyme-435 was reported to be used in the synthesis of polyamide or polyester from small molecules but did not produce any success with the case of cyclic peptides during our synthesis. Finally, the use of diamine cyclic peptide from the second approach and monofunctionalized carboxylic acid with a succinate linker was examined (third approach) using EDC and DMAP as classical peptide coupling reagents. The third approach afforded the tricyclic peptide, possibly due to the availability of four extra carbon atoms as a linker in the lysine side chain, which enhanced activation of the COOH group followed by conjugation to the diamine of the cyclic peptide.

### 3.2. Biological Activity

**Figure 2:**
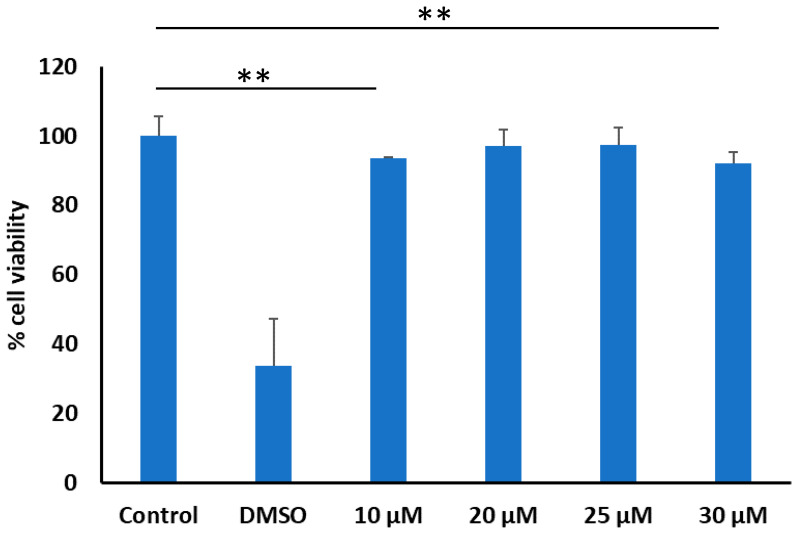
Cytotoxicity of tricyclic peptide **20** in MDA-MB-231 cells (if *p* < 0.01 then **). DMSO (35% *v/v*) was used as the positive control. The compound was used in the aqueous solution.

The cytotoxicity of tricyclic peptide **20** was determined by the MTS assay against breast cancer cell lines (MDA-MB-231). Tricyclic peptide **20** showed more than 97% cell viability in MDA-MB-231 cell lines up to a concentration of 25 μM, which slightly decreased to 92% at 30 μM (Figure 2). Thus, further cell-based assays were conducted at 25 μM.

**Figure 3:**
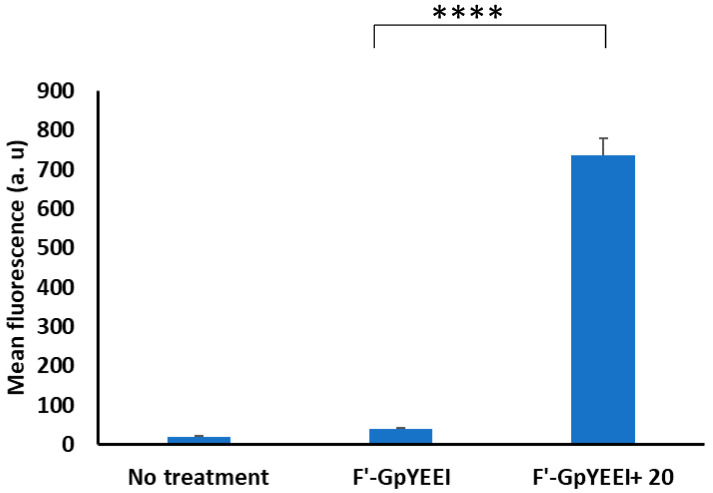
The cellular uptake of fluorescently labeled G-(pTyr)-Glu-Glu-Ile (F′-GpYEEI) using tricyclic peptide **20** in the MDA-MB-231 cells (if *p* < 0.0001 then ****). Untreated cells are shown on the left.

Phosphopeptides contain negatively-charged phosphate moieties, which restrict the peptides to cross the cellular membranes [36,37]. The molecular transport of these valuable probes into cellular systems presents a great advantage for the study of phosphoprotein-protein interactions as well as protein phosphorylation and dephosphorylation. The potential of tricyclic peptide **20** for the delivery of fluorescently labeled (F’)-Gly-(pTyr)-Glu-Glu-Ile (F’-GpYEEI) was analyzed with flow cytometry. The percentage of fluorescently positive cells exceeded 98%, while non-treated cells or phosphopeptide alone were less than 0.6%. In terms of mean fluorescence, the cellular uptake was enhanced at least by 18 times in the presence of tricyclic peptide **20**, suggesting the role of the latter as a successful delivery tool (Figure 3). The delivery efficiency of the tricyclic peptide was compared with monocyclic peptide [WR]_5_. However, the newly developed tricyclic peptide was found to be less efficient than the monomer. This could be due to the steric hindrance of the tricyclic peptide, which could restrict its binding affinity toward the phosphopeptide.

**Figure 4:**
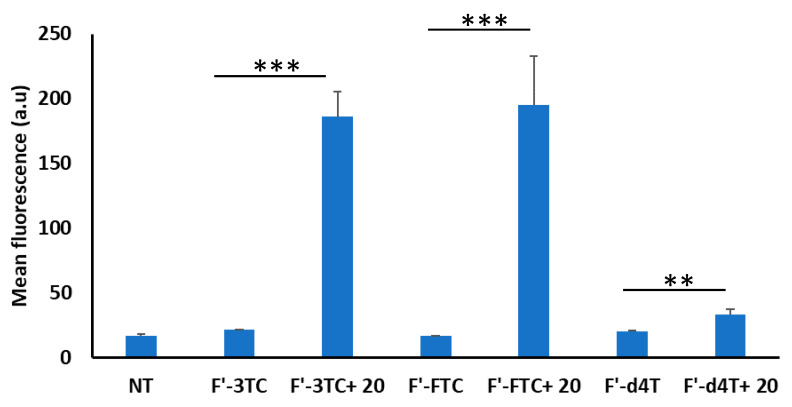
The cellular uptake of fluorescently labeled anti-HIV drugs (F′-FTC, F′-3TC, and F′-d4T) in the presence of tricyclic peptide **20** in MDA-MB-231 cells (if *p* < 0.01 then **, if *p* < 0.001 then ***).

To study the application of the newly synthesized tricyclic peptide **20** as a molecular transporter, the cellular uptake of three different fluorescently labeled anti-HIV drugs, lamivudine (3TC), emtricitabine (FTC), stavudine (d4T) (F′-3TC, F′-FTC and F′-d4T) was examined in the presence and absence of the tricyclic peptide **20** using MDA-MB-231 cell line (Figure 4). After 4 h of incubation at 37 °C, the cells were washed with PBS followed by treatment with trypsin. The cellular uptake was monitored with flow cytometry. The data demonstrated exhibited significantly greater fluorescence signals in the cells incubated with the F′-3TC and F′-FTC-loaded with the tricyclic peptide **20**, compared to those treated with the fluorescently-labeled compounds alone, indicating that the uptake of these two compounds is promoted by the tricyclic peptide. For instance, the cellular internalization in the presence of the peptide was approximately nine times higher for F′-3TC and almost twelve times higher for F′-FTC, which is significantly higher than the previously reported monocyclic peptide [WR]_5_ [13]. Interestingly, there was no significant increase in the mean fluorescence of the cells in the case of F′-d4T (less than twofold difference), although the percentage of fluorescently positive cells exceeded 98%. The lower cellular uptake of d4T as compared to 3TC or FTC could be speculated based on interactions of the pyrimidine ring of drugs with the phospholipid bilayer and tricyclic peptide **20**. This may be attributed to the presence of the amino group in the pyrimidine ring of 3TC and FTC as compared to d4T. More interactions possibly occur with the tricyclic peptide; therefore higher cellular uptake was achieved for FTC and 3TC.

**Figure 5:**
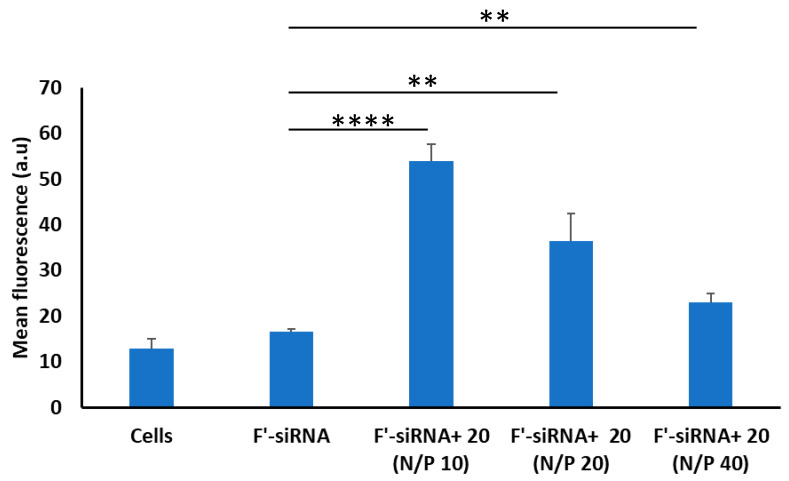
The cellular uptake of fluorescently-labeled siRNA in the presence of tricyclic peptide 20 in MDA-MB-231 cells (if *p* < 0.01 then **, if *p* < 0.0001 then ****).

siRNA has been shown as an alternative therapeutic approach in cancer treatment. One major obstacle for this approach has been the poor cellular internalization of the double-stranded RNA, which has anionic and hydrophilic character [38]. Different strategies have been used for siRNA delivery, including the use of synthesized cyclic peptides after complex formation with fluorescently labeled siRNA. Tricyclic peptide **20** showed 3.3-fold improvements in siRNA internalization compared to siRNA alone at the lowest peptide:siRNA ratio N/P 10, which progressively decreased with an increase in the N/P ratio (Figure 5) in the MDA-MBA-231 cell line. At a 20:1 ratio, the cellular internalization of siRNA decreased, and at 40:1 ratio, there was no major difference in siRNA uptake compared to free siRNA. Both of them did not show any statistical difference to the control group (NT). The result suggests that at a lower N/P ratio, the peptide shows appreciable efficiency in siRNA delivery; however, at higher N/P ratio, the efficiency is decreased. This may be attributed to the possible intermolecular hydrogen bonding among the arginine residues at high concentration, which reduces the available cationic charges in the peptide; thus, loading of anionic siRNA on peptides is decreased.

As a part of the innate immune system, peptides possess a broad-spectrum antimicrobial activity [39,40,41]. Cyclic peptides, in particular, are considered as an emerging class of antimicrobial peptides against antibiotic-resistant pathogens [42,43]. *c*[R_4_W_4_] is a cyclic amphiphilic peptide exhibited potent antibacterial activity against MRSA (MIC 4 µg/mL) and *E. coli* (MIC 16 µg/mL) and showed improved antibacterial activity when co-administered with tetracycline [23,24]. The structure-antibacterial activity of a series of *c*[R_4_W_4_] peptides revealed the requirement of R and W amino acids [24]. Thus, we decided to perform the antibacterial activity of tricyclic peptide **20**. The tricyclic peptide **20** exhibited modest antibacterial activity against *MRSA*, *Pseudomonas aeruginosa, Klebsiella pneumoniae*, and *E. Coli* with MIC values in the range of 64–128 µg/mL, which was significantly lower than *c*[R_4_W_4_] (Table 1). These data were not unexpected since the peptide contained alternative R and W residues rather than blocks of hydrophobic and positively charged residues on opposite sides, like *c*[R_4_W_4_], required for a more amphiphilic property to generate potent antibacterial activity.

## 4. Conclusions

We designed and synthesized tricyclic [(WR)_4_]_3_ after two unsuccessful synthetic strategies using Fmoc/tBu solid-phase synthesis. In the first approach, the intermolecular coupling of monfunctional cyclic peptides did not produce conjugated peptide, while in the second approach, the intermolecular coupling of diamine or dicarboxylic acid failed to produce a bicyclic peptide. However, by reacting protected diamine cyclic peptide **9** with a mono carboxylic-succinate-cyclic peptide **17**, tricyclic peptide **20** was synthesized. The evaluation of the antibacterial activity of tricyclic peptide showed modest activity against MRSA, *Pseudomonas aeruginosa, Klebsiella pneumoniae,* and *E. coli* with MIC values of 64–128 µg/mL. Tricyclic peptide **20** was also evaluated for molecular transporter activity after it was found to be nontoxic up to 30 µM in the MDA-MB-231. Tricyclic peptide **20** enhanced the cellular uptake of phosphopeptide F’-GpYEEI by 18-fold as compared to the uptake F’-GpYEEI alone. Similarly, the uptake of antiviral drugs (d4T, 3TC, and FTC) was enhanced in the presence of tricyclic peptide **20** by 1.9–12-fold as compared to the drug alone. Tricyclic peptide **20** also internalized a fluorescent-labeled siRNA into the MDA-MB-231 cells by 3.3-fold at N/P ratio of 10 as compared to siRNA alone. Further optimization in the design and application of tricyclic peptide will be required to access the full potential of these multivalent compounds in the biological system. Our data also reflect the potential of the tricyclic or polycyclic peptides in the area of drug delivery.

## Figures and Tables

**Table 1:** Antibacterial activity of Tricyclic peptide **20**.

Compound Name	MIC (µg/mL) #			
	MRSA (LAC Clone)	*Klebsiella pneumoniae* (ATCC BAA 1705)	*Pseudomonas aeruginosa* (ATCC 27883)	*E. coli* ATCC 25922)
**Meropenem**	4	32	1	8
***c*** **[R_4_W_4_]**	4	32	64	16
**Tricyclic Peptide 20**	64	128	128	64

# Experiments were performed in triplicate.

## Supplementary Materials

The following are available online at https://www.mdpi.com/1999-4923/12/9/842/s1, MALDI spectra of intermediate and synthesized compounds. Figure S1: Preparatory RP-HPLC profile of tricyclic peptide **20**, Figure S2: Analytical RP-HPLC profile of tricyclic peptide **20**, Figure S3: Analytical RP-HPLC profile of peptide **7**, Figure S4: Analytical RP-HPLC profile of peptide **10**, Figure S5: Analytical RP-HPLC profile of peptide **16**, Figure S6: Analytical RP-HPLC profile of peptide **18**, Figure S7: Analytical RP-HPLC profile of crude peptide **12**.
