# Supplementary material for: Click-Free Synthesis of a Multivalent Tricyclic Peptide as a Molecular Transporter
Source: Pharmaceutics. 2020 Sep 3;12(9):842. doi: 10.3390/pharmaceutics12090842 (PMC7558522; doi:10.3390/pharmaceutics12090842)
Supplement: Supplementary file 1 [file pharmaceutics-12-00842-s001.pdf]

pharmaceutics

Supplementary Materials: Click-Free Synthesis of a Multivalent Tricyclic Peptide as a Molecular Transporter

Sumit Kumar, Dindyal Mandal, Shaima Ahmed El-Mowafi, Saghar Mozaffari, Rakesh Kumar Tiwari and Keykavous Parang

Content

Representative MALDI Spectra and HPLC of Intermediate and Synthesized Compounds (Page 3-15).

Peptide #2

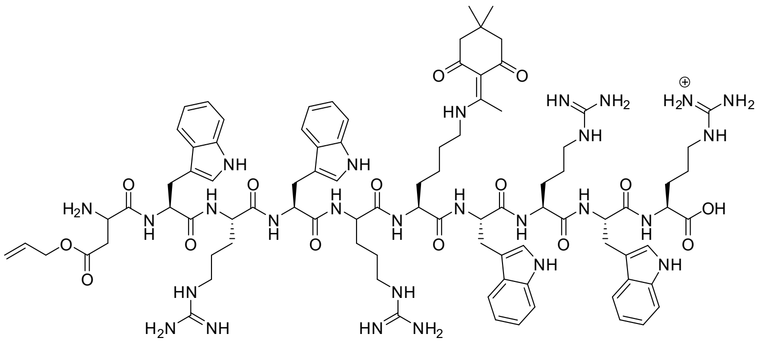

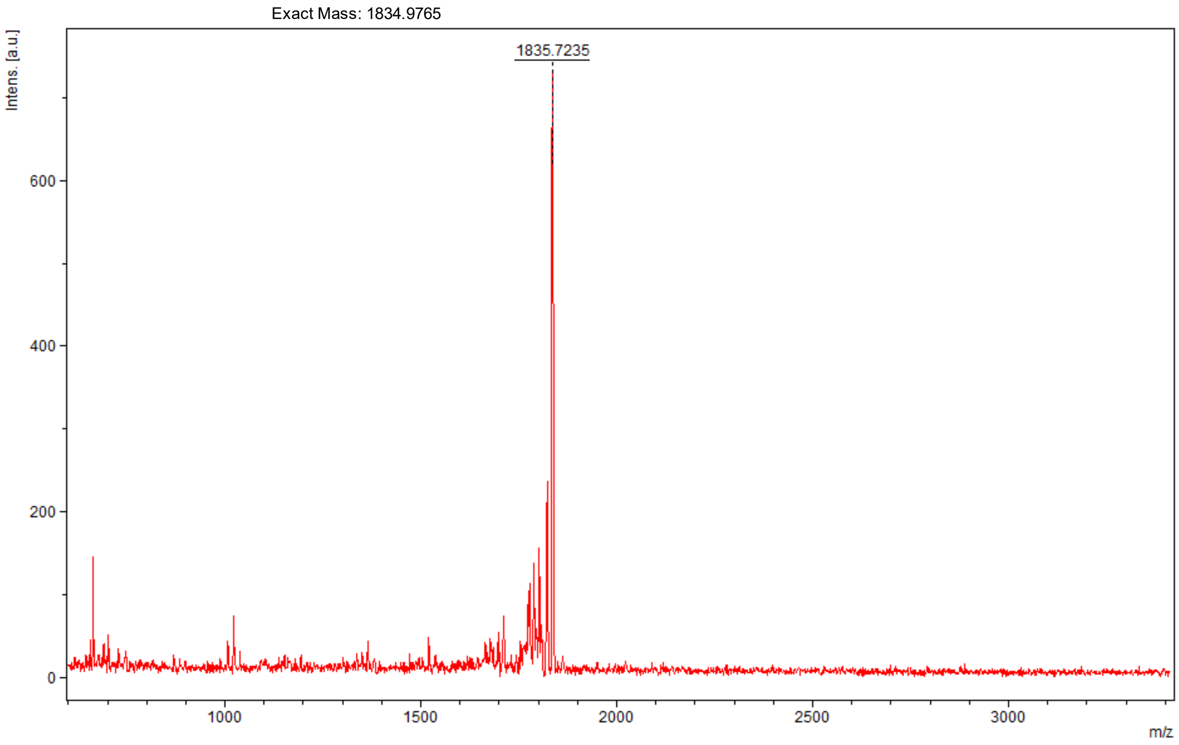

Peptide #3

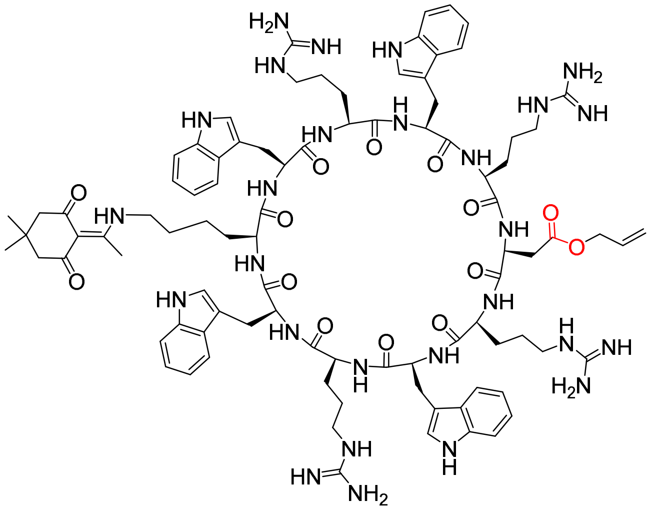

Exact Mass: 1815.9586

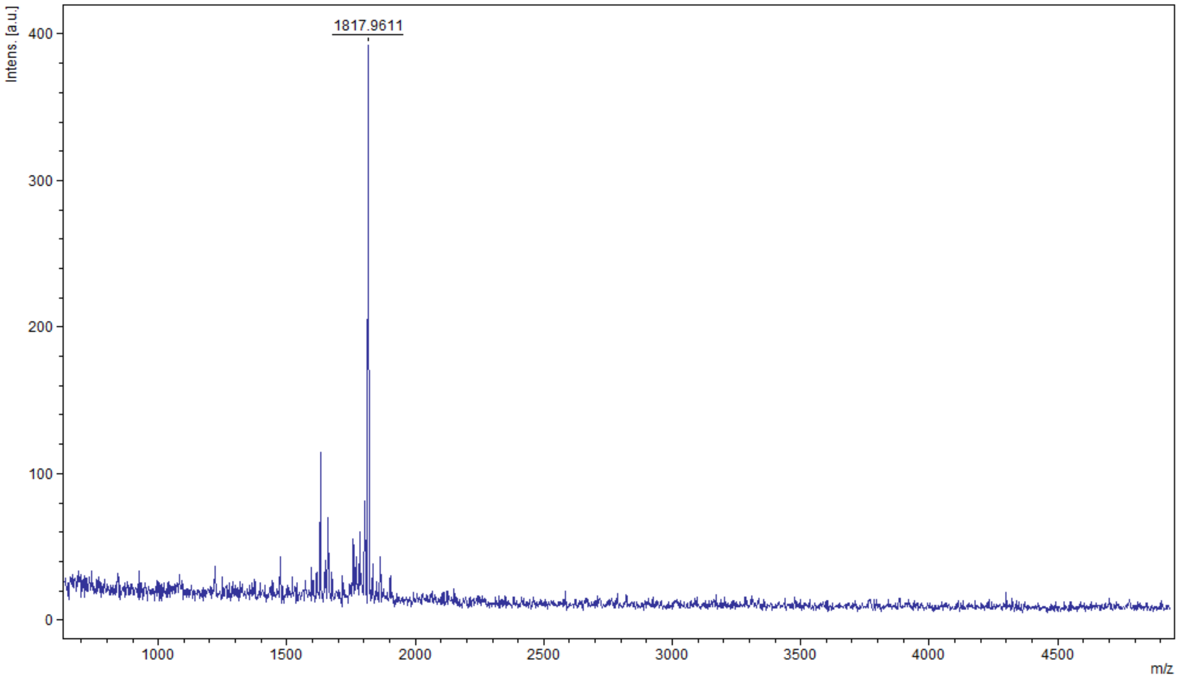

Peptide #4

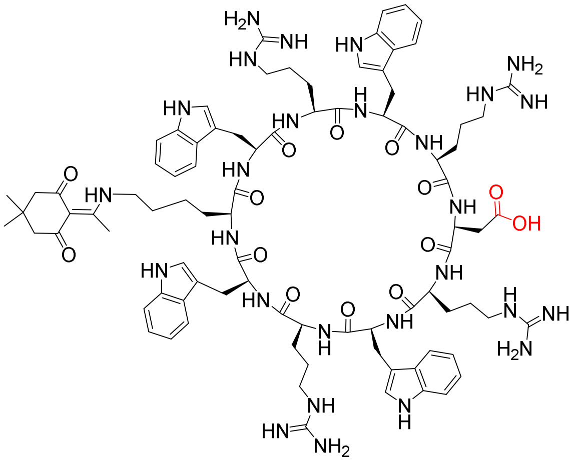

Exact Mass: 1775.9273

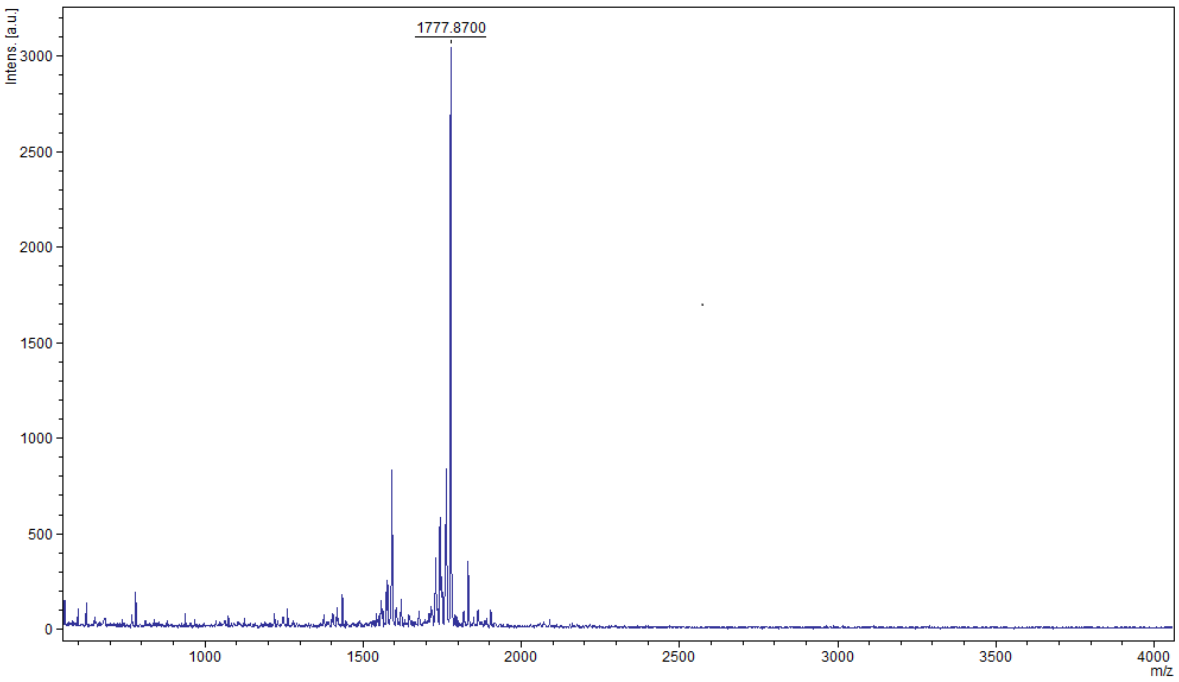

Peptide #7

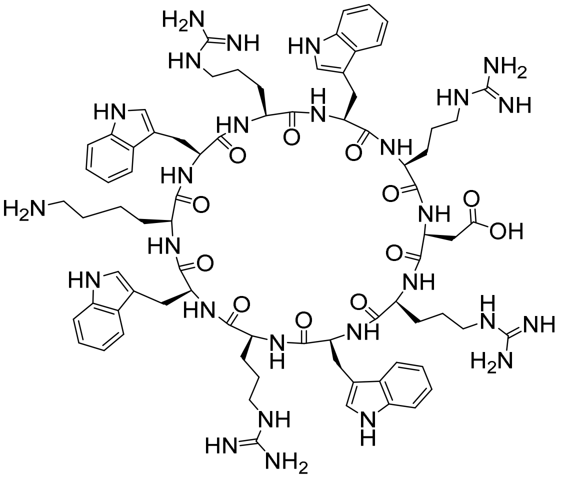

Exact Mass: 1611.8436

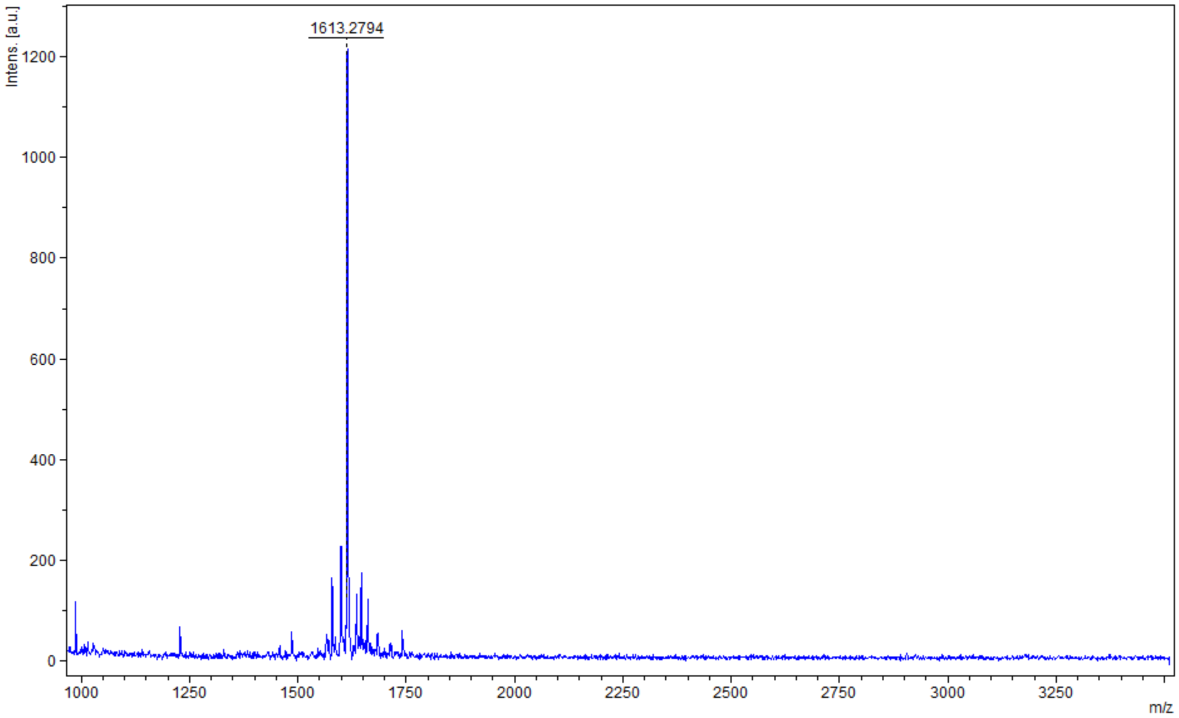

Peptide #8

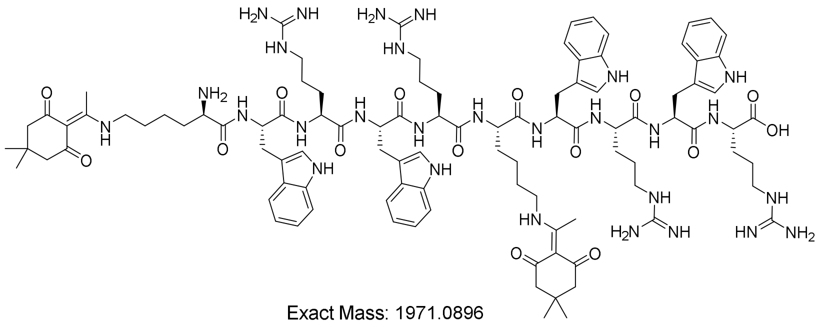
Exact Mass: 1971.0896

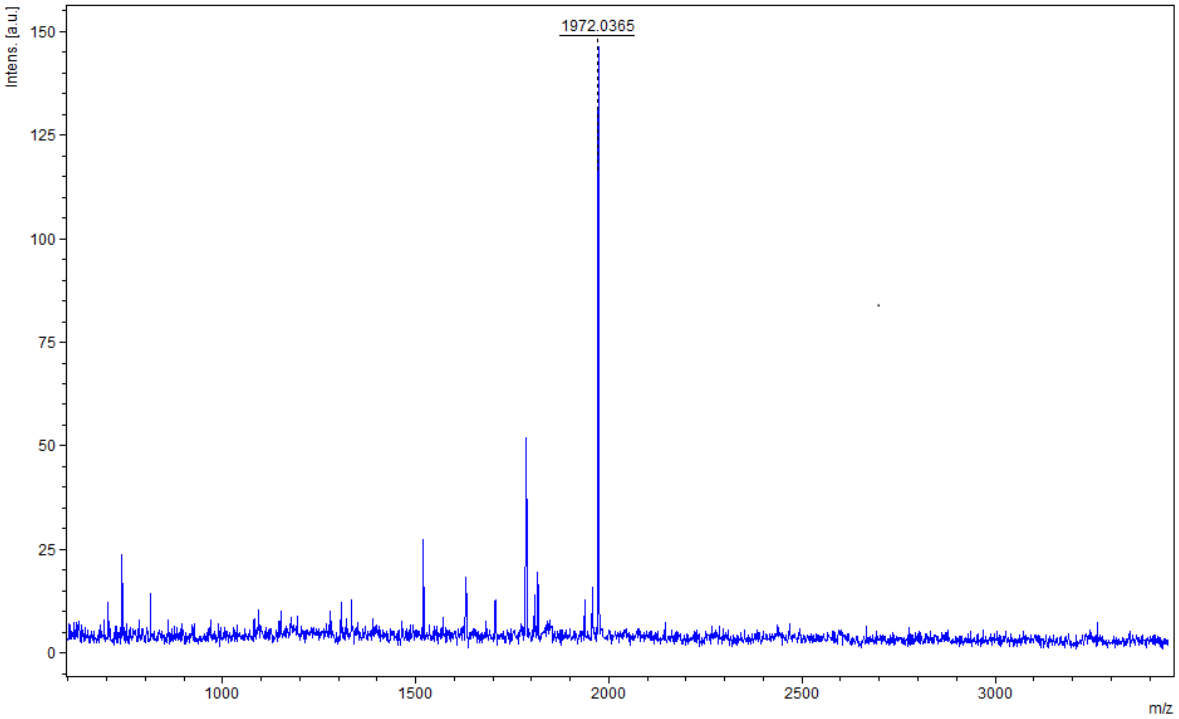
1972.0365
Intens. [a.u.]
150
125
100
75
50
25
0
1000
1500
2000
2500
3000
m/z

Peptide #8 after Dde deprotection.

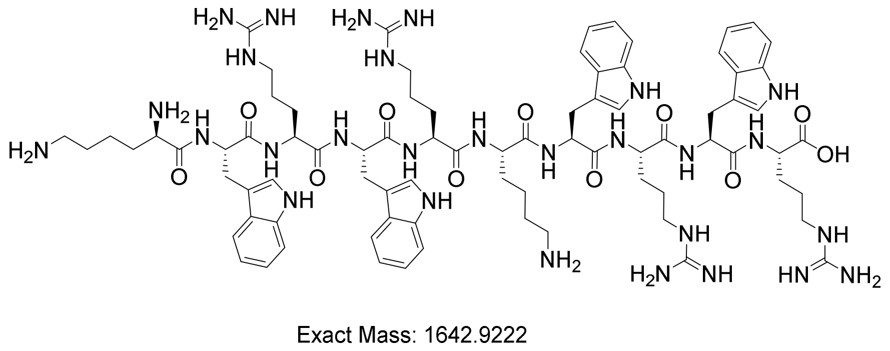

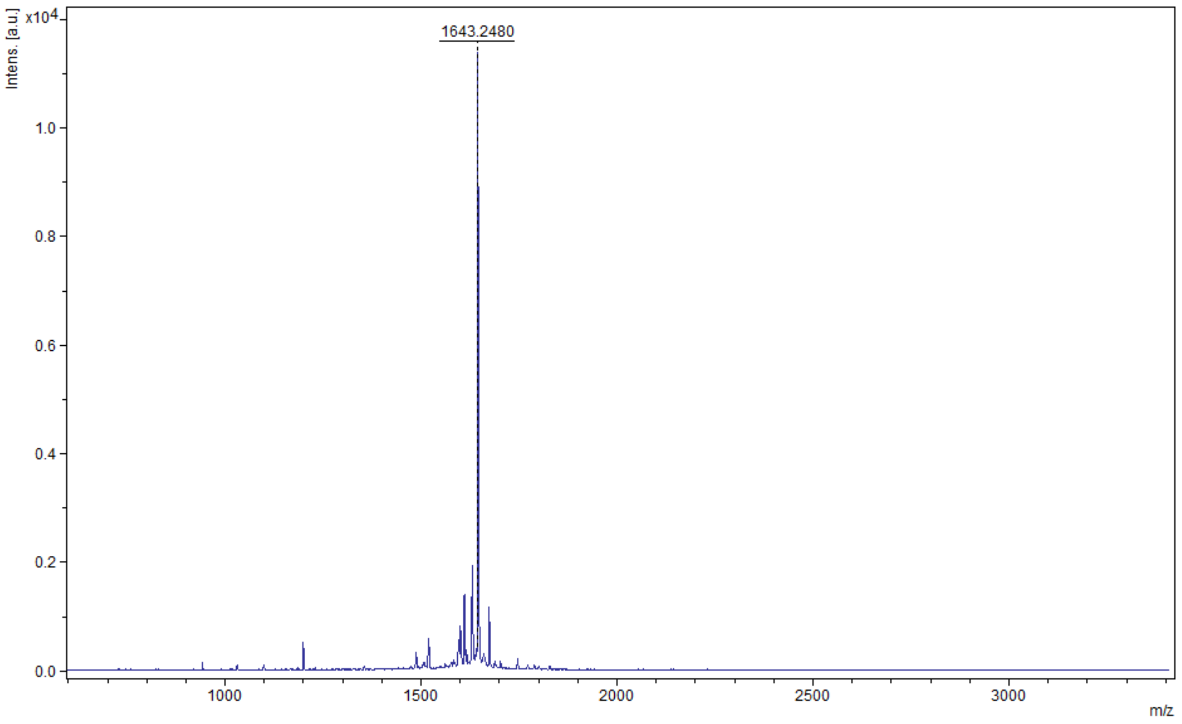

Peptide # 9/10 with Dde

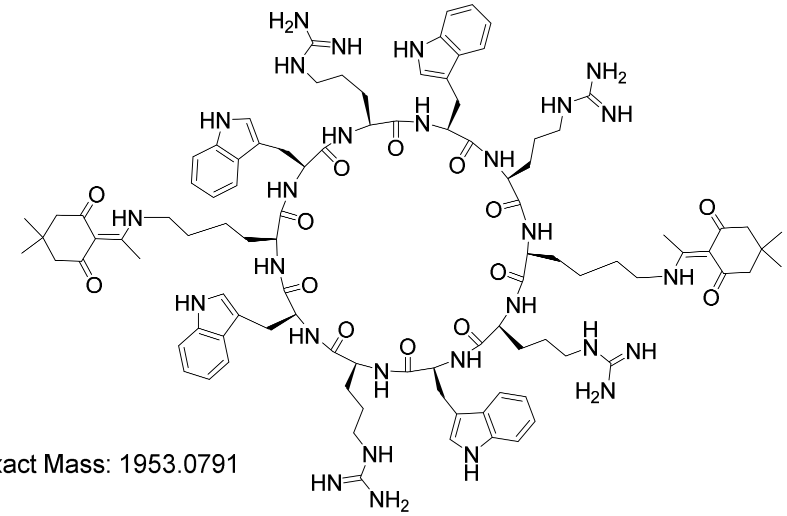

Exact Mass: 1953.0791

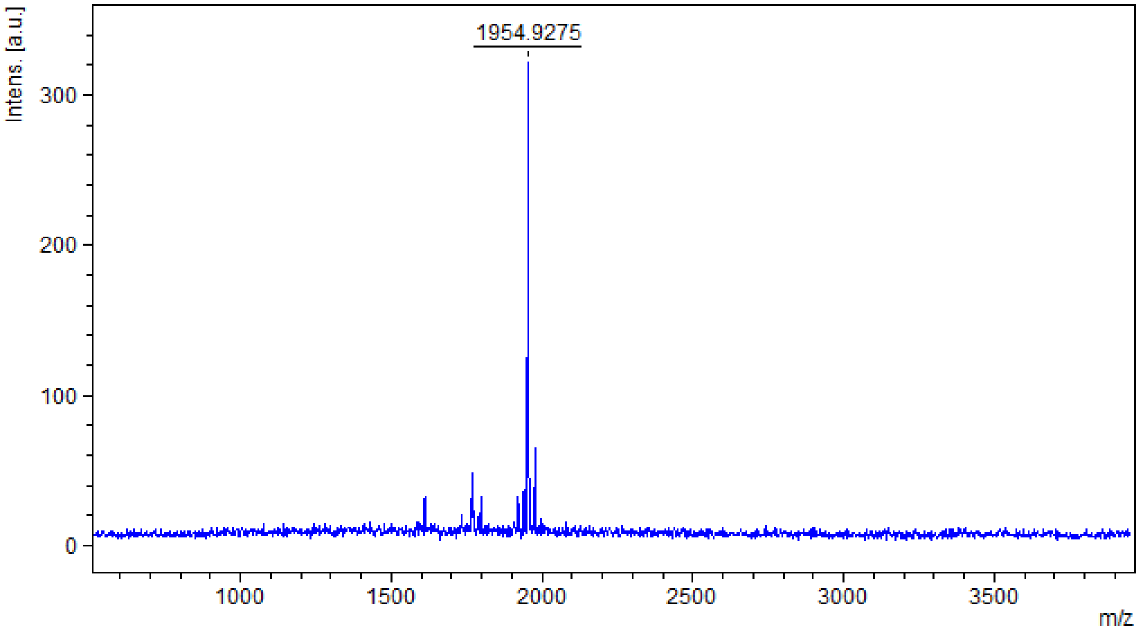

Peptide #10

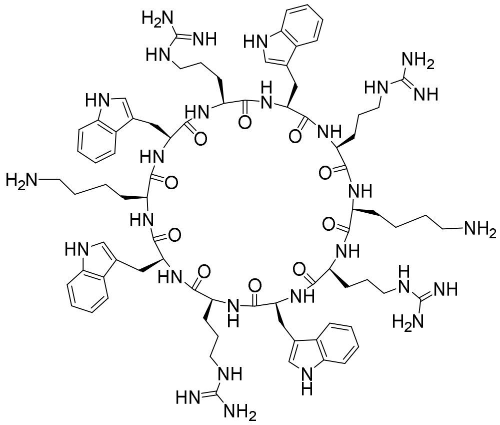

Exact Mass: 1624.9116

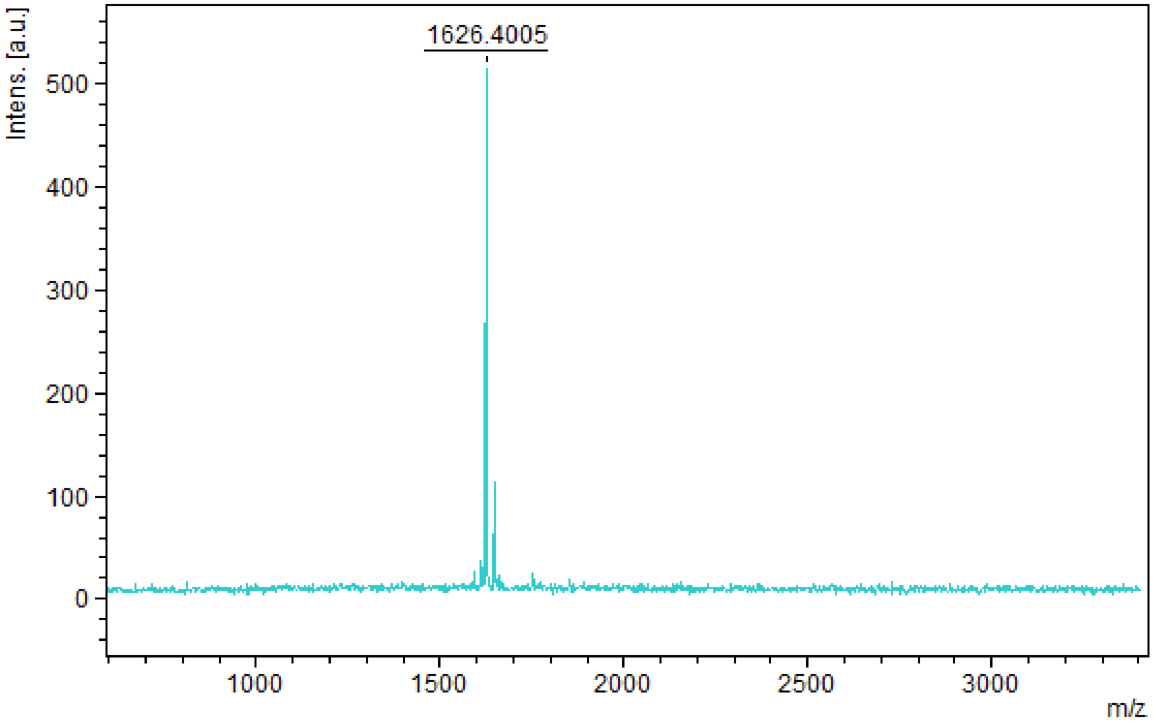

Peptide #12

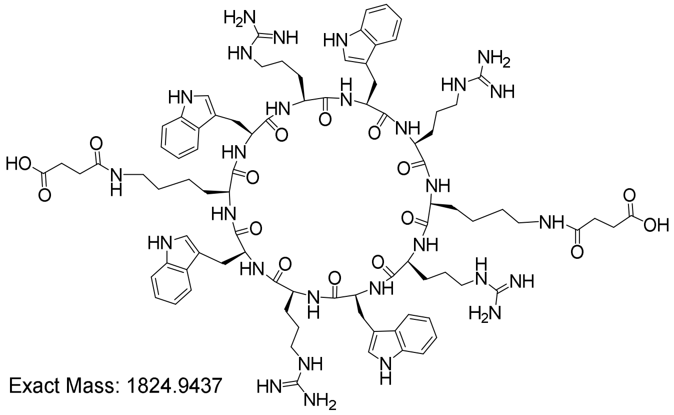
Exact Mass: 1824.9437

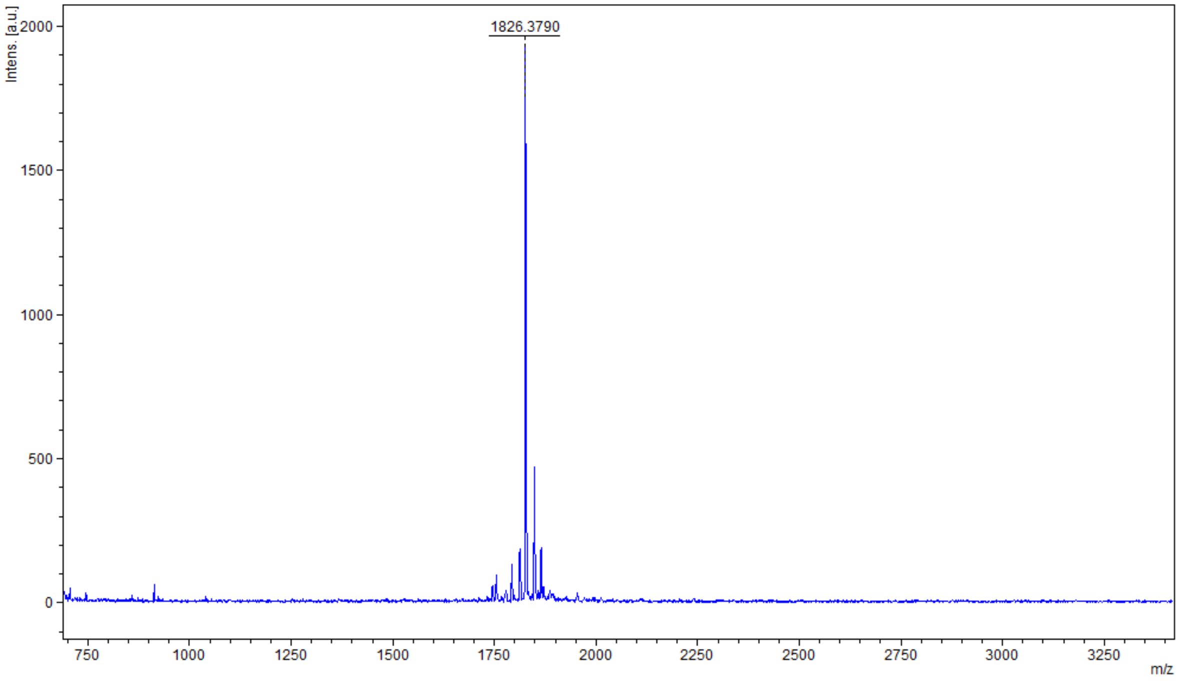
1826.3790
Intens. [a.u.]
2000
1500
1000
500
0
750
1000
1250
1500
1750
2000
2250
2500
2750
3000
3250
m/z

Peptide #13

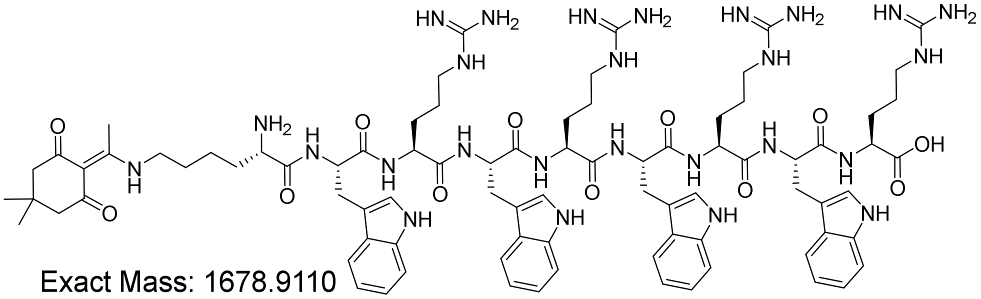

Exact Mass: 1678.9110

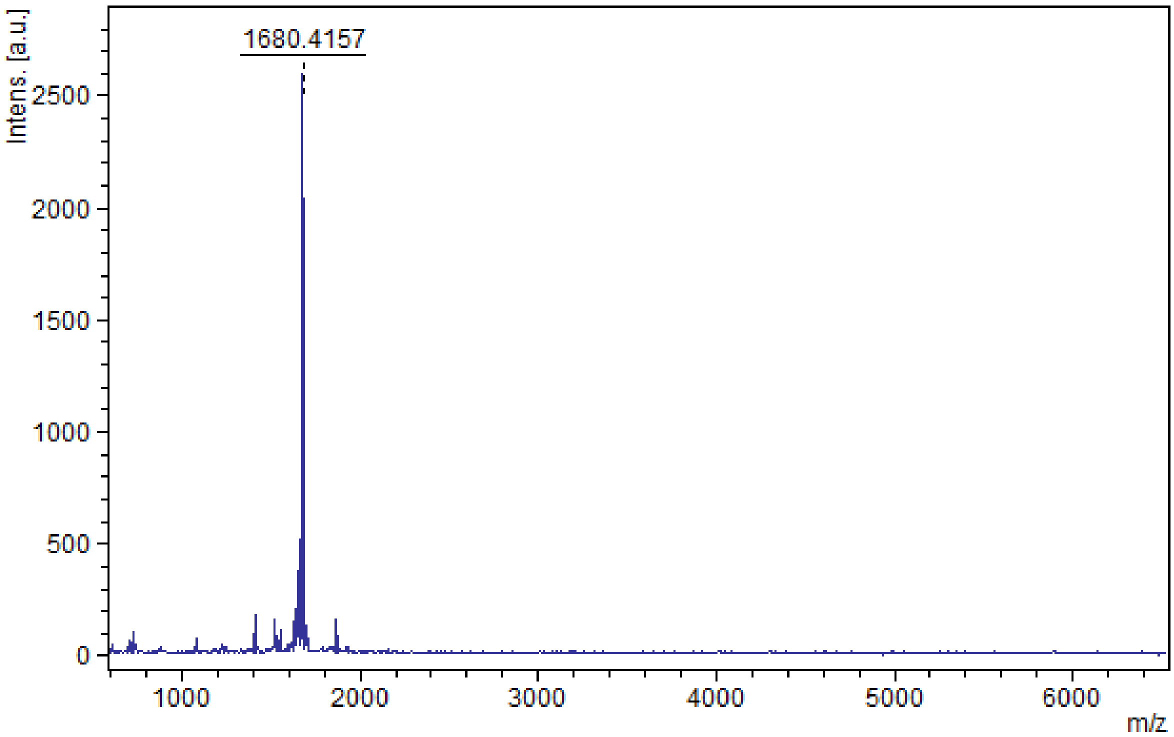

Peptide #16

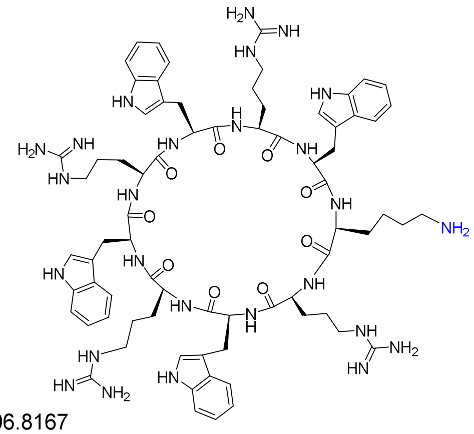

Exact Mass: 1496.8167

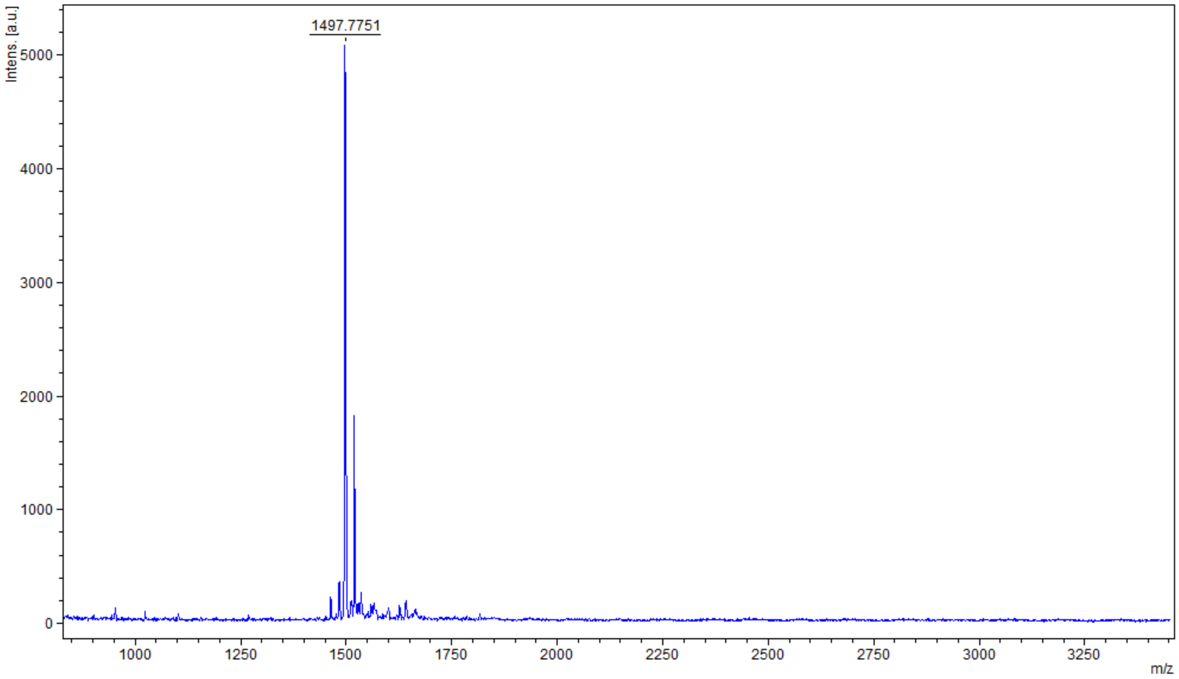

Peptide #18

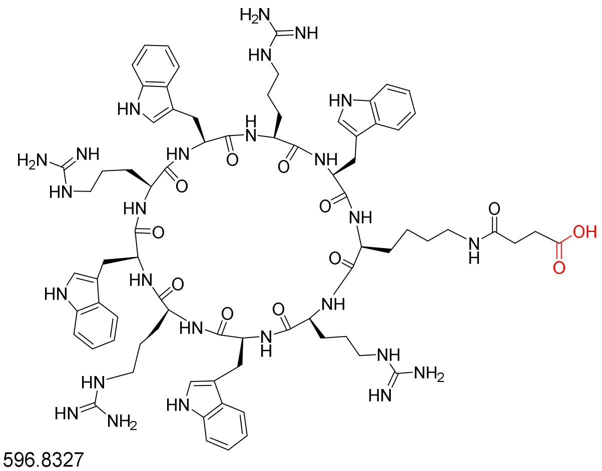

Exact Mass: 1596.8327

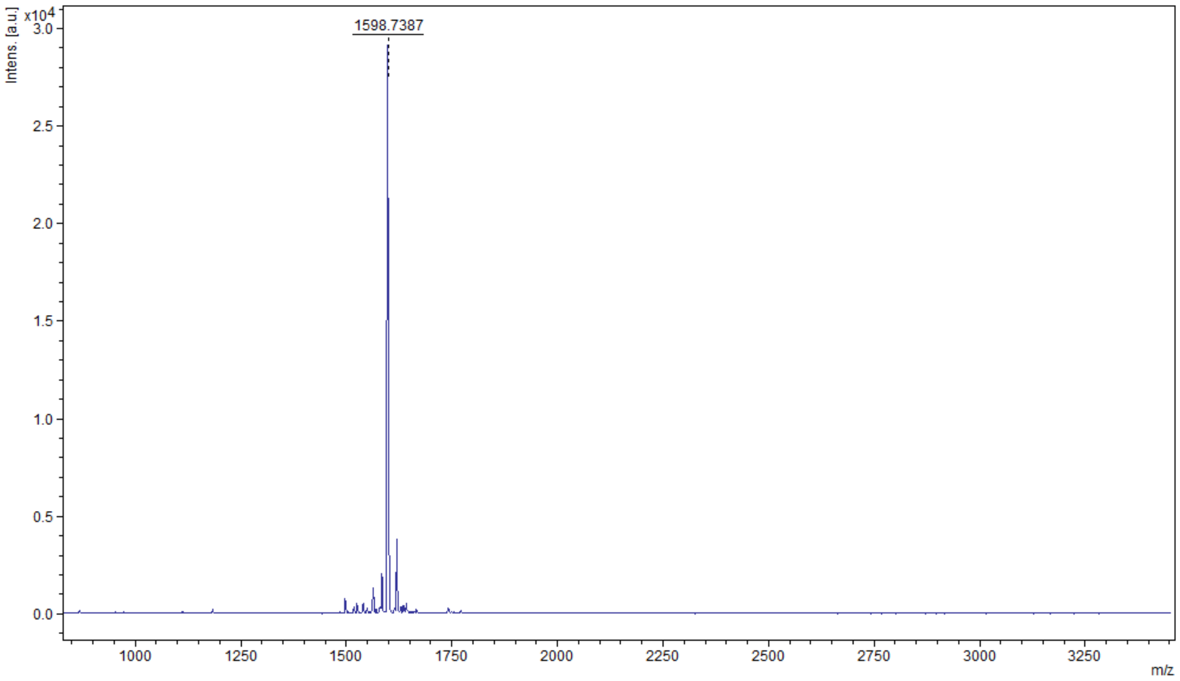

Peptide #20

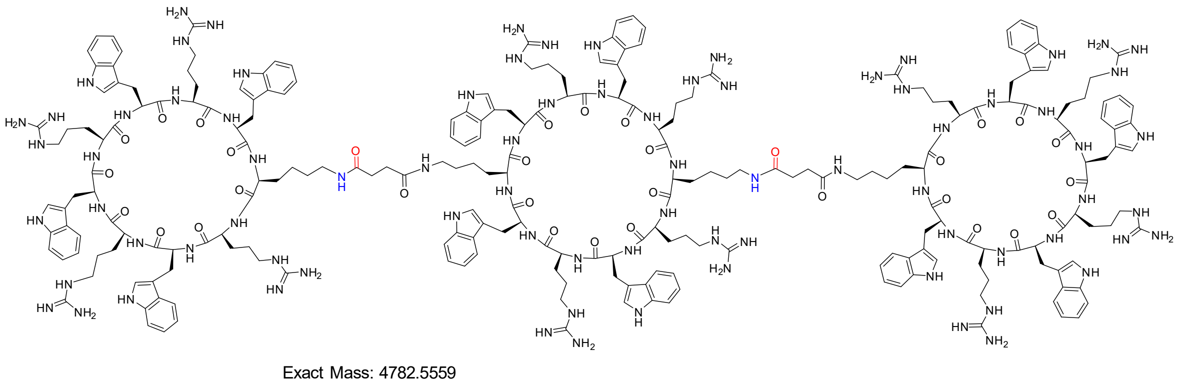

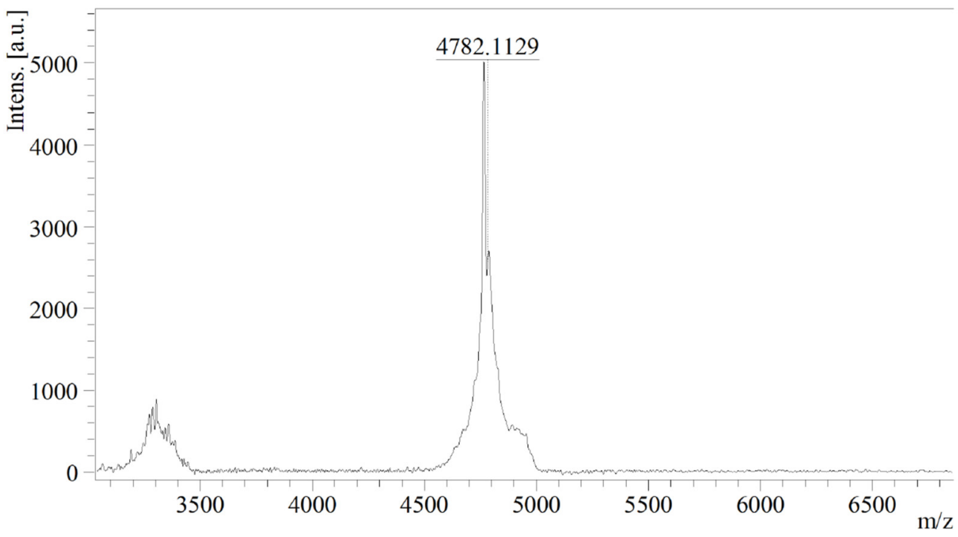

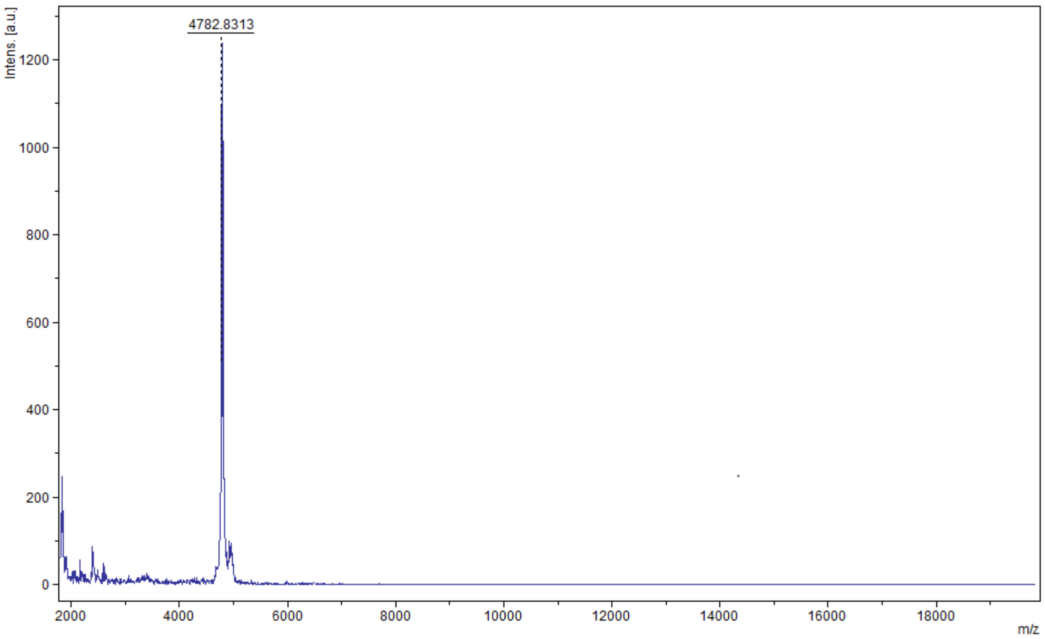

HPLC of compound 20

Tricyclic peptide **20** was purified using reverse phase-high performance liquid chromatography (RP-HPLC) using Hitachi L-2455 system (Canby, OR, USA) with a C18 Phenomenex column (Prodigy, 10 µm, 2.1 cm × 25 cm), at a flow rate of 10 mL/min with the detection at 214 nm using a gradient of 0-100% acetonitrile (0.1% trifluoracetic acid (TFA)) and water (0.1% TFA) over 60 min.

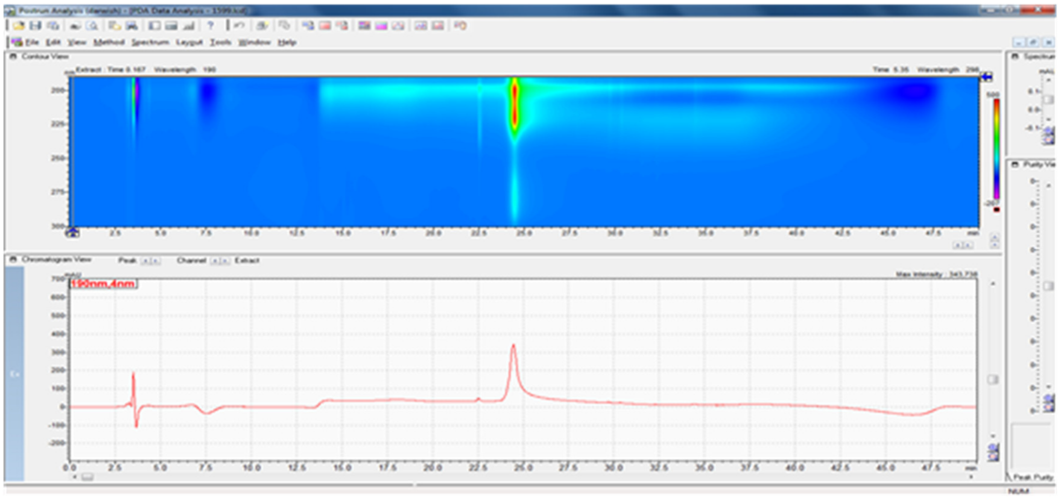

Figure S1. Preparatory RP-HPLC profile of tricyclic peptide **20**.

The analytical HPLC (HITACHI LaChrom ULTRA) was run with a gradient system of acetonitrile and water with 0.1% TFA (v/v) using a reversed-phase VyDAC column (218TP54, 5 µm, 4.60 mm x 150 mm) and flow rate of 1 mL/min with the detection at 214 nm.

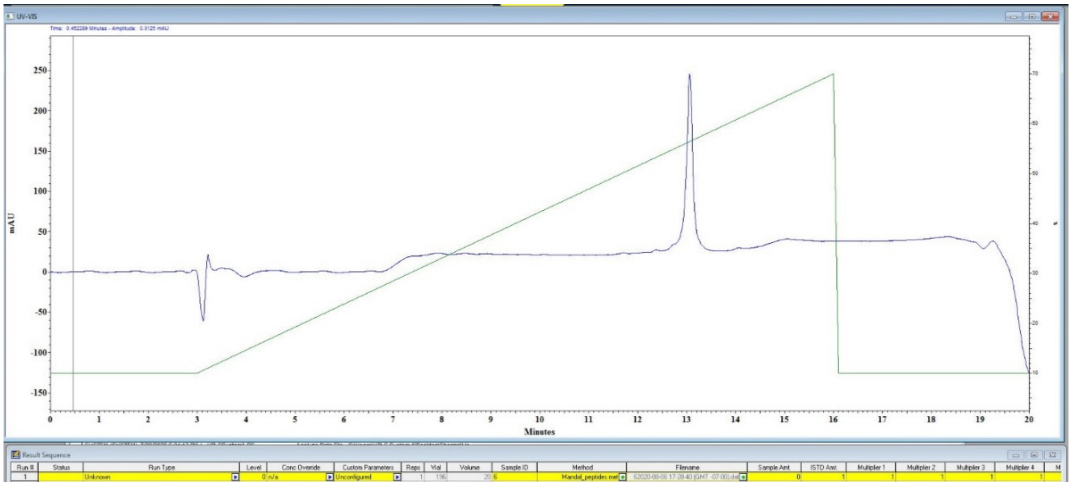

Figure S2. Analytical RP-HPLC profile of tricyclic peptide **20**.

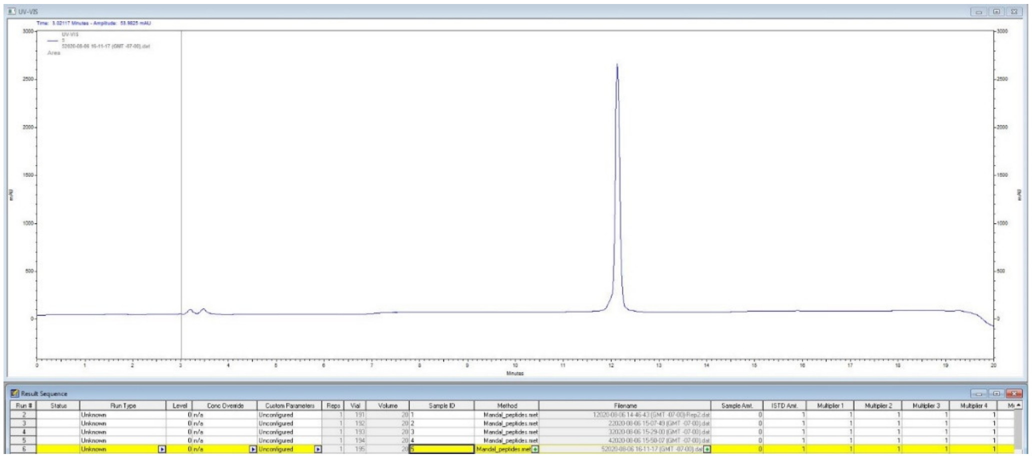

Figure S3. Analytical RP-HPLC profile of peptide **7**.

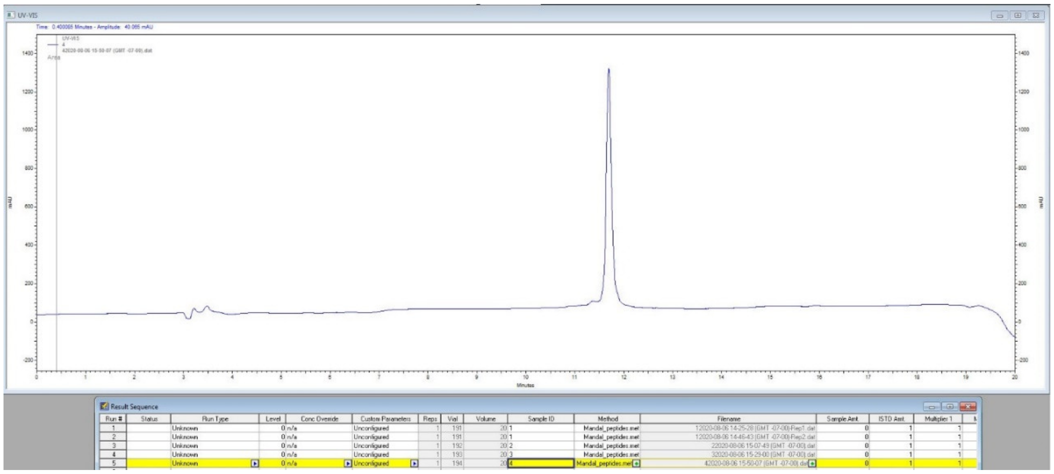

Figure S4. Analytical RP-HPLC profile of peptide **10**.

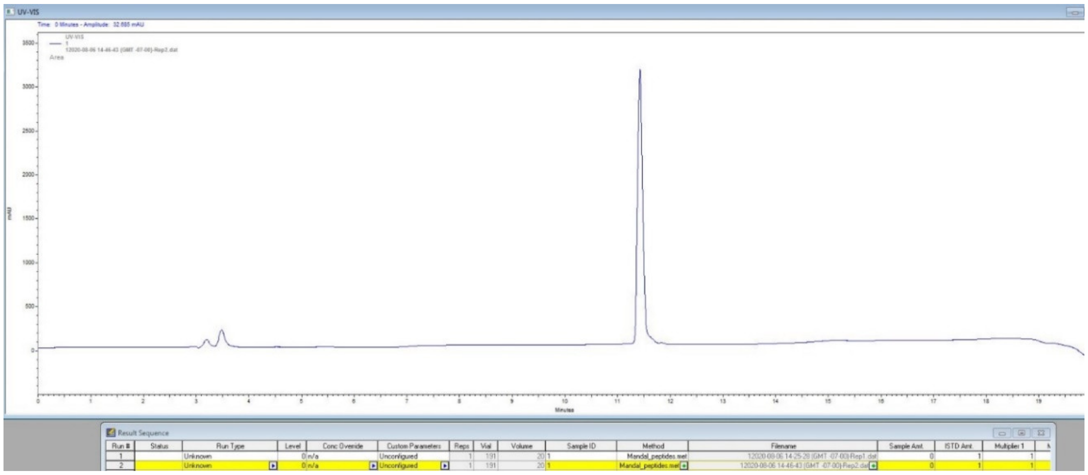

Figure S5. Analytical RP-HPLC profile of peptide **16**.

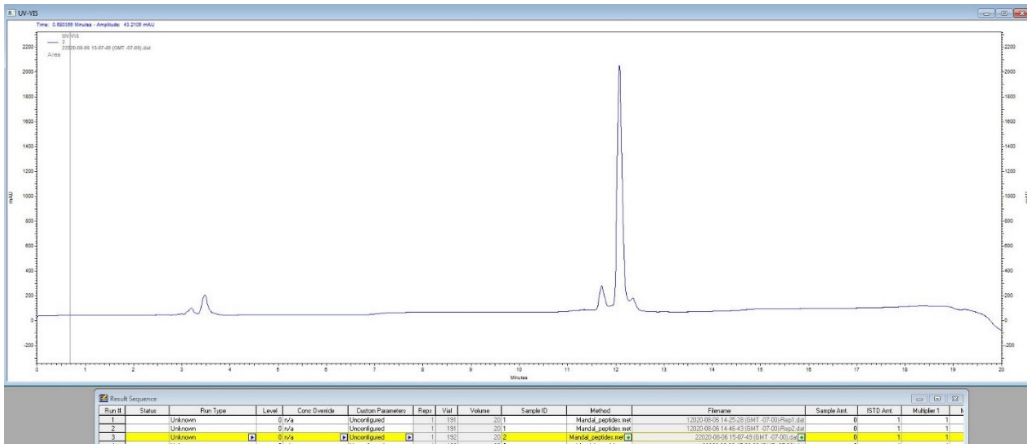

Figure S6. Analytical RP-HPLC profile of peptide **18**.

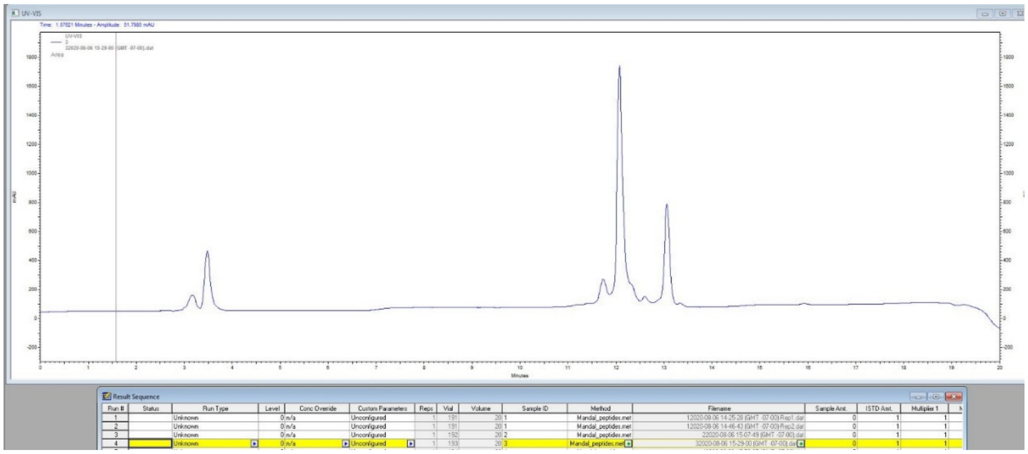

Figure S7. Analytical RP-HPLC profile of crude peptide **12**.
